# Calderón problem for nonlocal viscous wave equations: Unique determination of linear and nonlinear perturbations

**DOI:** 10.1007/s13398-025-01822-0

**Published:** 2026-01-09

**Authors:** Philipp Zimmermann

**Affiliations:** 1 Department of Mathematics, ETH Zurich, Zürich, Switzerland; 2 Departament de Matemàtiques i Informàtica, Universitat de Barcelona, Barcelona, Spain

**Keywords:** Fractional Laplacian, Viscous wave equations, Nonlinear PDEs, Inverse problems, Runge approximation, Nemytskii operators, Primary 35R30, Secondary 26A33, 42B37

## Abstract

The main goal of this article is to study a Calderón type inverse problem for certain viscous nonlocal wave equations. We show that the partial Dirichlet to Neumann map uniquely determines on the one hand linear perturbations and on the other hand homogeneous nonlinearities *f*(*u*) whenever the latter satisfy a certain growth assumption. As a preliminary step we discuss the well-posedness in each case, where for the nonlinear setting we invoke the implicit function theorem after establishing the differentiability of the associated Nemytskii operator *f*(*u*). In the linear case we establish a Runge approximation theorem in $L^2(0,T;\widetilde{H}^{s}(\Omega ))$, which allows us to uniquely determine potentials that belong only to $L^{\infty }(0,T;L^p(\Omega ))$ for some $1<p\le \infty$ satisfying suitable restrictions. In the nonlinear case, we first derive an appropriate integral identity and combine this with the differentiability of the solution map around zero to show that the nonlinearity is uniquely determined by the Dirichlet to Neumann map. To make this linearization technique work, it is essential that we have a Runge approximation in $L^2(0,T;\widetilde{H}^s(\Omega ))$ instead of $L^2(\Omega _T)$ at our disposal.

## Introduction

In recent years, many different inverse problems for nonlocal partial differential equations (PDEs) have been studied in the literature. The very first work in this area was the article [11] by Ghosh, Salo and Uhlmann. They showed that the nonnegative potential $q\in L^{\infty }(\Omega )$ in the *fractional Schrödinger equation*

1.1 $$\begin{aligned} ((-\Delta )^s+q)u=0\text { in }\Omega \end{aligned}$$

is uniquely determined by the *(partial) Dirichlet to Neumann (DN) map*

$$\begin{aligned} \Lambda _q f= (-\Delta )^s u_f|_{W_2}, \quad f\in C_c^{\infty }(W_1), \end{aligned}$$

for arbitrary fixed measurement sets $W_1,W_2\subset \Omega _e$. Here, $\Omega \subset {\mathbb {R}}^n$ is a bounded domain with exterior $\Omega _e={\mathbb {R}}^n\setminus \overline{\Omega }$, $0<s<1$ and $u_f:{\mathbb {R}}^n\rightarrow {\mathbb {R}}$ denotes the unique solution to (1.1) with exterior data $u_f|_{\Omega _e}=f$. An essential analytical tool in their work is the so-called *unique continuation property (UCP)* of the fractional Laplacian. Roughly speaking, the UCP can be phrased as follows:

If $u:{\mathbb {R}}^n\rightarrow {\mathbb {R}}$ satisfies $(-\Delta )^su=u=0$ in an open set $V\subset {\mathbb {R}}^n$, then $u\equiv 0$.

Its proof depends on the famous *Caffarelli–Silvestre (CS) extension* [4] of the fractional Laplacian, which allows to characterize the fractional Laplacian of *u* as the Neumann data of the solution *U* to the degenerate elliptic PDE

$$\begin{aligned} {{\,\textrm{div}\,}}\left( y^{1-2s}\nabla U(x,y)\right) =0 \text { in }{\mathbb {R}}^{n+1}_+ \end{aligned}$$

with Dirichlet data $U(x,0)=u$ on $\partial {\mathbb {R}}^{n+1}_+$. Solutions to such equations, having $A_2$ Muckenhoupt weights as coefficients, have already been studied a long time ago in the celebrated work [7] by Fabes, Kenig and Serapioni. Let us note that CS type extensions are only available for a restricted classes of nonlocal operators as discussed in more detail by Kwaśnicki, Mucha and Stinga, Torrea in [16] and [35], respectively. Based on this fact, in subsequent research articles in this field, the main focus was put on nonlocal inverse problems for equations of the form

1.2 $$\begin{aligned} L_K u+Q(u)=0\text { in }\Omega , \end{aligned}$$

where $L_K$ is an elliptic (with potentially variable coefficients) nonlocal operator having the UCP and *Q* is a possibly nonlinear function of *u*. Prototypical results in this field show that the DN map associated with equation (1.2) uniquely determines the function *Q* (see e.g. [2, 3, 10, 12, 13, 30, 32]). Additionally, there are works in which the authors have attempted to recover the coefficients *K*, on which the nonlocal operator $L_K$ may depend, from the associated DN map. Examples of such nonlocal operators *L* include the fractional powers of elliptic second order operators $\textrm{L}^s_{\sigma }=\left( -{{\,\textrm{div}\,}}(\sigma \nabla )\right) ^s$ and the fractional conductivity operator $L^s_{\gamma }$. Both examples fall into the class of elliptic integro-differential operators, denoted by $\mathcal {L}_0$, which consists of all operators $L_K$ that can be written in strong form as

$$\begin{aligned} L_Ku(x)=\text {p.v.}\int _{{\mathbb {R}}^n}K(x,y)(u(x)-u(y))\,dy, \end{aligned}$$

where the kernel *K*(*x*, *y*) satisfies

1.3 $$\begin{aligned} K(x,y)=K(y,x)\quad \text {and}\quad \frac{c}{|x-y|^{n+2s}}\le K(x,y)\le \frac{C}{|x-y|^{n+2s}}. \end{aligned}$$

The second condition in (1.3) simply means that the kernel *K*(*x*, *y*) is comparable to that of the fractional Laplacian $(-\Delta )^s$ (see Sect. 2 for more details on the fractional Laplacian). In [9, Sect. 2.3] it is shown that for every uniformly elliptic coefficient $\sigma \in C^{\infty }({\mathbb {R}}^n)$, the nonlocal operator $\textrm{L}_{\sigma }^s=\left( -{{\,\textrm{div}\,}}(\sigma \nabla )\right) ^s$ belongs to $\mathcal {L}_0$, and its kernel is given by

$$ \textrm{K}^s_{\sigma }(x,y)=\frac{1}{\Gamma (-s)}\int _0^{\infty }p_t(x,y)\frac{dt}{t^{1+s}}, $$

where $\Gamma$ denotes Euler’s Gamma function and $p_t$ is the symmetric heat kernel associated with the elliptic operator $\textrm{L}_{\sigma }=-{{\,\textrm{div}\,}}(\sigma \nabla )$. We note that the bounds in (1.3) for $\textrm{K}^s_{\sigma }$ follow from the fact that the heat kernel $p_t$ can be controlled from both sides by Gaussians; that is,

$$ c (4\pi t)^{-n/2} e^{-\frac{|x-y|^2}{4\alpha t}}\le p_t(x,y)\le C (4\pi t)^{-n/2} e^{\frac{|x-y|^2}{4\beta t}} $$

for some constants $c,C,\alpha ,\beta >0$. To see that $L^s_{\gamma }\in \mathcal {L}_0$, we recall that the kernel of the fractional conductivity operator $L^s_{\gamma }$ is given by

$$ K^s_{\gamma }(x,y)=C_{n,s}\frac{\gamma ^{1/2}(x)\gamma ^{1/2}(y)}{|x-y|^{n+2s}} $$

where $C_{n,s}>0$ is a suitable normalization constant and $\gamma :{\mathbb {R}}^n\rightarrow {\mathbb {R}}$ is a uniformly elliptic function. The works [31, 33] provide affirmative answers to the question of whether the kernels $K^s_{\gamma }$ and $\textrm{K}^s_{\sigma }$ can be recovered from the associated DN maps. Let us remark that there are only very few results on the simultaneous recovery of the leading order coefficient *K* and the lower order perturbation *Q*. For this type of questions, we may point the interested reader to the works [27, 39], and [22].

Later on these studies were extended to the parabolic setting (e.g., [19–21, 23, 26]). Recently, Kow, Lin and Wang studied in [15] a Calderón type inverse problem related to the *nonlocal wave equation*^1^

1.4 $$\begin{aligned} {\left\{ \begin{array}{ll} \left( \partial _t^2 +(-\Delta )^s+q\right) u=0 & \text { in }\Omega _T\\ u=\varphi & \text { in }(\Omega _e)_T,\\ u(0)=0,\quad \partial _t u(0)=0 & \text { in }\Omega \end{array}\right. } \end{aligned}$$

with $0<s<1$, $q=q(x)\in L^{\infty }(\Omega )$ and showed under suitable assumptions on the domains that:

(i) *Runge approximation:* Any $v\in L^2(\Omega _T)$ can be approximated arbitrarily well in $L^2(\Omega _T)$ by solutions $u_{\varphi }|_{\Omega _T}$ of (1.4), where $\varphi \in C_c^{\infty }(W_T)$ for some fixed measurement set $W\Subset \Omega _e$.
(ii) *Unique determination:* Let $q_1,q_2\in L^{\infty }(\Omega )$ be two potentials and denote by $\Lambda _{q_j}$ its DN map related to (1.4), i.e.
  $$ \Lambda _{q_j}\varphi =\left. (-\Delta )^su_{\varphi }\right| _{(\Omega _e)_T}. $$
  If one has
  $$ \left. \Lambda _{q_1}\varphi \right| _{(W_2)_T}=\left. \Lambda _{q_2}\varphi \right| _{(W_2)_T} $$
  for all $\varphi \in C_c^{\infty }((W_1)_T)$, where $W_1,W_2\subset \Omega _e$ are two fixed measurement sets, then one has
  $$ q_1(x)=q_2(x)\text { in }\Omega . $$

Let us point out that in contrast to the elliptic (see [32]) or the parabolic case (see [23]), it is not known whether the Runge approximation for the nonlocal wave equation (1.4) holds in $L^2(0,T;\widetilde{H}^s(\Omega ))$^2^. Hence, the following question remains open.

### Question 1

Is the Runge set

$$ \mathcal {R}_W=\{u_{\varphi }-\varphi \,;\,\varphi \in C_c^{\infty }(W_T)\}, $$

where the notation $u_{\varphi }$ is as above, dense in $L^2(0,T;\widetilde{H}^s(\Omega ))$?

The main obstruction in proving such a Runge approximation is the low regularity of solutions to the equation

$$ {\left\{ \begin{array}{ll} \left( \partial _t^2 +(-\Delta )^s+q\right) u=F & \text { in }\Omega _T \\ u=0 & \text { in }(\Omega _e)_T, \\ u(0)=0,\quad \partial _t u(0)=0 & \text { in }\Omega , \end{array}\right. } $$

when *F* only belongs to the space $L^2(0,T;H^{-s}(\Omega ))$ instead of $L^2(\Omega _T)$. This phenomenon already appears in the local case $s=1$ (see [29, Chapter 11]). On the other hand, a main difference between the local and nonlocal wave equation (1.4) is that in the latter case the equation does not have a finite speed of propagation, which in turn relies on the UCP of the fractional Laplacian.

Because of the lack of such a density result the techniques of this article cannot be directly adapted to the nonlinear nonlocal wave equation and it is an open question, whether the DN map related to the *nonlinear nonlocal wave equation*

$$\begin{aligned} {\left\{ \begin{array}{ll} \left( \partial _t^2 +(-\Delta )^s\right) u+f(u)=0 & \text { in }\Omega _T\\ u=\varphi & \text { in }(\Omega _e)_T,\\ u(0)=0,\quad \partial _t u(0)=0 & \text { in }\Omega \end{array}\right. } \end{aligned}$$

uniquely determines suitable nonlinearities *f*. Here and below, *f*(*u*) denotes the Nemytskii operator associated to a Carathéodory function $f:\Omega \times {\mathbb {R}}\rightarrow {\mathbb {R}}$, that is

$$\begin{aligned} f(u)(x,t)=f(x,u(x,t)). \end{aligned}$$

In this article, we study a Calderón type inverse problem for linear and nonlinear perturbations of the *nonlocal viscous wave equation*

1.5 $$\begin{aligned} {\left\{ \begin{array}{ll} \left( \partial _t^2 +(-\Delta )^s\partial _t+(-\Delta )^s\right) u=0 & \text { in }\Omega _T\\ u=\varphi & \text { in }(\Omega _e)_T,\\ u(0)=0,\quad \partial _t u(0)=0 & \text { in }\Omega . \end{array}\right. } \end{aligned}$$

The terminology used for this equation is discussed in the next section. More concretely, this means that we study the problems

1.6 $$\begin{aligned} {\left\{ \begin{array}{ll} \left( \partial _t^2 +(-\Delta )^s\partial _t+(-\Delta )^s+q\right) u=0 & \text { in }\Omega _T\\ u=\varphi & \text { in }(\Omega _e)_T,\\ u(0)=0,\quad \partial _t u(0)=0 & \text { in }\Omega \end{array}\right. } \end{aligned}$$

and

1.7 $$\begin{aligned} {\left\{ \begin{array}{ll} \left( \partial _t^2 +(-\Delta )^s\partial _t+(-\Delta )^s\right) u+f(u)=0 & \text { in }\Omega _T\\ u=\varphi & \text { in }(\Omega _e)_T,\\ u(0)=0,\quad \partial _t u(0)=0 & \text { in }\Omega \end{array}\right. } \end{aligned}$$

and aim to uniquely recover the potential *q* and nonlinearity *f* under suitable assumptions from the related DN maps

$$\begin{aligned} \begin{aligned} \Lambda _q \varphi&=\left. ((-\Delta )^su_\varphi +(-\Delta )^s\partial _t u_{\varphi })\right| _{(\Omega _e)_T}\\ \Lambda _{f} \varphi&=\left. ((-\Delta )^sv_\varphi +(-\Delta )^s\partial _t v_{\varphi })\right| _{(\Omega _e)_T}, \end{aligned} \end{aligned}$$

where $u_\varphi , v_\varphi$ are the unique solutions to (1.6) and (1.7), respectively. Let us remark that in contrast to the results in [15] the potential in (1.6) is allowed to vary in time and is not necessarily bounded in space.

Finally, let us note that in fact one can construct unique solutions to the linear nonlocal wave equations by first considering solutions $u_\varepsilon$ to the nonlocal viscous wave equation with loss term $\varepsilon (-\Delta )^s\partial _t$ and then passing to the limit $\varepsilon \rightarrow 0$ (see [5, Chapter XVIII]).

### Nonlocal viscous wave equations and related models

The main goal of this section is to motivate the terminology for equation (1.5) and to discuss related models.

First of all let us recall that the initial and boundary value problem for the *viscous wave equation* is given by

1.8 $$\begin{aligned} {\left\{ \begin{array}{ll} \left( \frac{1}{c^2}\partial _t^2 +\tau (-\Delta )\partial _t+(-\Delta )\right) u=0 & \text { in }\Omega _T\\ u=\varphi & \text { on }\partial \Omega _T,\\ u(0)=0,\quad \partial _t u(0)=0 & \text { in }\Omega , \end{array}\right. } \end{aligned}$$

which emerges in acoustics to describe the propagation of sound in a viscous fluid. The quantity *u* represents the sound pressure, *c* the speed of propagation and $\tau$ the relaxation time, which can be calculated as

$$ \tau = \frac{4\mu }{3\rho _0c^2} $$

with $\mu$ being the shear bulk viscosity coefficient, which has been measured for many fluids, and $\rho _0$ is the static density. The term $\tau (-\Delta )u$ corresponds physically to an additional loss term. If we formally put $c=\tau =1$ and replace the Laplacian by the fractional Laplacian $(-\Delta )^s$, then we arrive at the problem (1.5).

Next, we describe a time fractional generalization of the problem (1.8) and a generalization of (1.5).

1. The first model we introduce reads as follows:
  $$\begin{aligned} {\left\{ \begin{array}{ll} \left( \frac{1}{c^2}\partial _t^2 +\beta \tau ^\beta (-\Delta )\partial _t^\beta +(-\Delta )\right) u=0 & \text { in }\Omega _T\\ u=\varphi & \text { on }\partial \Omega _T,\\ u(0)=0,\quad \partial _t u(0)=0 & \text { in }\Omega . \end{array}\right. } \end{aligned}$$
  Here $\partial _t^{\beta }$ denotes a fractional time derivative. Such operators naturally arise when interpolating between Hooke’s law—which relates the strain and stress in an elastic solid—and Newton’s law for fluids, which describes the linear relationship between stress and the strain rate in an ideal viscous fluid. For further information on this model, we refer to [37, 38] and the references therein.
2. An immediate generalization of the model (1.5) is
  1.9 $$\begin{aligned} {\left\{ \begin{array}{ll} \left( \partial _t^2 +(-\Delta )^{s_1}\partial _t+(-\Delta )^{s_2}\right) u=0 & \text { in }\Omega _T\\ u=\varphi & \text { in }(\Omega _e)_T,\\ u(0)=0,\quad \partial _t u(0)=0 & \text { in }\Omega , \end{array}\right. } \end{aligned}$$
  where two fractional Laplacians of different orders $s_1$ and $s_2$ appear. The special case $s_1 = \tfrac{1}{2}$ and $s_2 = 1$ has recently attracted interest and has been studied in the setting $\Omega = \mathbb {R}^2$, for example in [6, 14, 17]. In these works, the equation may also include a nonlinearity of the form $|u|^{p-1}u$ for $p>1$ or $u^k$ for $k \ge 2$. Such power-type nonlinearities have been extensively studied in the context of dispersive wave equations, such as the nonlinear wave equation or the nonlinear Schrödinger equation
  $$ i\partial _t u+\Delta u+f(u)=0\text { in }{\mathbb {R}}^n $$
  (see [36]). Inverse problems related to the model (1.9) are addressed in upcoming work of the author with Katya Krupchyk [18].

### Main results

In this section, we present our main results on the inverse problems for the nonlocal viscous wave equation with linear and nonlinear perturbations.

#### Theorem 1.1

(Uniqueness of linear perturbations) Let $\Omega \subset {\mathbb {R}}^n$ be a bounded Lipschitz domain, $T>0$ and $s>0$ a non-integer. Suppose that for $j=1,2$ we have given potentials $q_j\in L^1_{loc}(\Omega _T)$ such that

(i) $q_j\in L^{\infty }(0,T;L^p(\Omega ))$ for some $1\le p<\infty$ satisfying
  $$ {\left\{ \begin{array}{ll} n/s\le p\le \infty , & \, \text {if }\, 2s< n, \\ 2<p\le \infty , & \, \text {if }\, 2s= n, \\ 2\le p\le \infty , & \, \text {if }\, 2s > n, \end{array}\right. } $$
(ii) $t\mapsto \int _{\Omega }q_j(x,t)\varphi (x)\,dx\in C([0,T])$ for any $\varphi \in C_c^{\infty }(\Omega )$.

Furthermore, assume that $W_1,W_2\subset \Omega _e$ are given measurement sets such that the DN maps $\Lambda _{q_j}$ related to

$$ {\left\{ \begin{array}{ll} \left( \partial _t^2 +(-\Delta )^s\partial _t +(-\Delta )^s+q_j\right) u=0 & \text { in }\Omega _T \\ u=\varphi & \text { in }(\Omega _e)_T, \\ u(0)=0,\quad \partial _t u(0)=0 & \text { in }\Omega . \end{array}\right. } $$

satisfy

1.10 $$\begin{aligned} \Lambda _{q_1}\varphi =\Lambda _{q_2}\varphi \text { in }(W_2)_T \end{aligned}$$

for all $\varphi \in C_c^{\infty }((W_1)_T)$. Then there holds

$$\begin{aligned} q_1(x,t)=q_2(x,t)\text { in }\Omega _T. \end{aligned}$$

In Sect. 4.2, we first present the proof of Theorem 1.1 for time-reversal invariant potentials, based on a suitable integral identity. Throughout this article, we define the *time reversal* of a function $Q \in L^1_{\textrm{loc}}(V_T)$, where $V \subset \mathbb {R}^n$ is an arbitrary open set, by

$$\begin{aligned} Q^{\star }(x,t) = Q(x, T - t), \end{aligned}$$

and we say that $Q$ is *time-reversal invariant* if $Q^\star = Q$. We then establish the proof of Theorem 1.1 for general potentials.

Afterwards, in Sect. 5, we extend this approach to show that nonlinear perturbations $f(x,\tau )$ are uniquely determined by the DN map:

#### Theorem 1.2

(Uniqueness of nonlinear perturbations) Let $\Omega \subset {\mathbb {R}}^n$ be a bounded Lipschitz domain, $T>0$ and $s>0$ a non-integer. Suppose that for $j=1,2$ we have given nonlinearities $f_j:\Omega \times {\mathbb {R}}\rightarrow {\mathbb {R}}$ satisfying Assumption 3.4 with $0<r\le 2$ and $f_j$ is $r+1$ homogeneous in the second variable. Let $U^j_0\subset \widetilde{W}^s_{rest}((\Omega _e)_T),U^j_1\subset \widetilde{W}_{ext}(0,T;\widetilde{H}^s(\Omega ))$ be the neighborhoods of the origin provided by Theorem 3.8, which have the property that, for any $\varphi \in U^j_0$, the problem

1.11 $$\begin{aligned} {\left\{ \begin{array}{ll} (\partial _t^2 +(-\Delta )^s\partial _t +(-\Delta )^s)u+f_j(u)= 0& \text { in }\Omega _T\\ u=\varphi & \text { in }(\Omega _e)_T,\\ u(0)=0,\quad \partial _t u(0)=0 & \text { in }\Omega \end{array}\right. } \end{aligned}$$

admits a unique solution $u\in U_1^j$. Furthermore, assume that $W_1,W_2\subset \Omega _e$ are given measurement sets such that the DN maps $\Lambda _{f_j}$ related to (1.11) satisfy

1.12 $$\begin{aligned} \Lambda _{f_1}\varphi =\Lambda _{f_2}\varphi \text { in }(W_2)_T \end{aligned}$$

for all $\varphi \in U_0^1\cap U_0^2$ that are supported in $(W_1)_T$. Then there holds

1.13 $$\begin{aligned} f_1(x,\rho )=f_2(x,\rho )\text { for }x\in \Omega \text { and }\rho \in {\mathbb {R}}. \end{aligned}$$

#### Remark 1.3

Both uniqueness theorems can be extended to other nonlocal operators *L* instead of the fractional Laplacian as long as they satisfy appropriate structural assumptions and a corresponding UCP. For this purpose we recall the necessary tools to solve the forward problem in a general framework in Sect. 2.3. Note that this has been done in the case of elliptic nonlocal inverse problems in the work [32].

### Recent developments in inverse problems for nonlocal wave equations

Since the appearance of the present article, the author, in collaboration with Y.-H. Lin, S.-R. Fu, T. Tyni, and Y. Yu, has obtained several unique determination results for the Calderón problem associated with nonlocal wave equations of the form

1.14 $$\begin{aligned} (\partial _t^2 + \gamma \partial _t + \lambda (-\Delta )^s \partial _t + (-\Delta )^s)u + f(u) = 0 \end{aligned}$$

and third order nonlocal wave equations of the form

1.15 $$\begin{aligned} (\partial _t^3 + \alpha \partial _t^2 + b(-\Delta )^s \partial _t + c(-\Delta )^s)u + f(u) = 0. \end{aligned}$$

(i) The works [24] and [8] establish the recovery of potentials $q$ and homogeneous nonlinearities $f$ in the nonlocal wave equation (1.14) with $\gamma = \lambda = 0$, as well as in the third order nonlocal wave equation (1.15), from the associated DN maps. Their proofs rely on the classical $L^2(\Omega _T)$ Runge approximation theorem for these equations.
(ii) In [25], an $L^2(0,T;\widetilde{H}^s(\Omega ))$ Runge approximation theory was developed for the nonlocal wave equation (1.14) with $\gamma = \lambda = 0$. This result was subsequently extended to the damped case $\gamma \ne 0$. Consequently, these works resolve Question 1 raised on p. 3. The key ingredient is the construction of so-called *very weak solutions*, which allow rough initial data and sources.
(iii) The improved Runge approximation results in (ii) allow for the unique determination of polyhomogeneous nonlinearities $f(x,u)$ whose growth at infinity exceeds the classes considered in earlier articles. Moreover, in [40] (for the equation (1.14) with $\lambda =0$), the damping coefficient $\gamma$ is permitted to vary over $\Omega$, and it is shown that the DN map simultaneously determines both the nonlinearity $f$ and the damping coefficient $\gamma$.
(iv) Finally, in [8] the nonlinearity $f$ in (1.15) is assumed to satisfy the same structural conditions as in the works above, but may additionally depend on the time variable $t$. The analysis developed there extends naturally to the other settings, and in particular Theorem 1.2 can be generalized to nonlinearities of the form $f(x,t,\tau )$. For simplicity, however, we confine ourselves in this article to the time-independent case.

### Organization of the article

This article is organized as follows. In Sect. 2, we introduce the functional analytic setup and in particular introduce the fractional Sobolev spaces, the fractional Laplacian and the Bochner Lebesgue spaces. Moreover, in the last paragraph of this section we discuss an abstract framework for solving some classes of second order in time PDEs. In Sect.  3, we study the well-posedness theory of the problems (1.6) and (1.7). The well-posedness of the nonlinear problem is achieved by invoking the implicit function theorem and to this end we study first in Sect. 3.2.1 the differentiability of the Nemytskii operator $u\mapsto f(u)$ (Lemma 3.7). Then in Sect. 4, after establishing the Runge approximation (Proposition 4.2) and a suitable integral identity (Lemma 4.3), we prove Theorem 1.1. Finally, in Sect. 5 we prove with the help of a suitable integral identity (Lemma 5.2) and the linearizatzion of the DN map the main theorem on the inverse problem for the nonlocal viscous wave equation with a nonlinear perturbation (Theorem 1.2).

## Preliminaries

In this section, we introduce several function spaces together with the fractional Laplacian, recall some important properties of this nonlocal operator and describe an abstract framework for solving some classes of second order in time PDEs.

### Fractional Sobolev spaces and fractional Laplacian

We denote by $\mathscr {S}({\mathbb {R}}^n)$ and $\mathscr {S}^{\prime }({\mathbb {R}}^n)$ Schwartz functions and tempered distributions respectively. We define the Fourier transform by

$$\begin{aligned} \mathcal {F}u(\xi ) := \hat{u}(\xi ):= \int _{{\mathbb {R}}^n} u(x)e^{-\textrm{i}x \cdot \xi } \,dx. \end{aligned}$$

By duality it can be extended to the space of tempered distributions and will again be denoted by $\mathcal {F}u = \widehat{u}$, where $u \in \mathscr {S}^{\prime }({\mathbb {R}}^n)$, and we denote the inverse Fourier transform by $\mathcal {F}^{-1}$.

Given $s\in {\mathbb {R}}$, the fractional Sobolev space $H^{s}({\mathbb {R}}^{n})$ is the set of all tempered distributions $u\in \mathscr {S}^{\prime }({\mathbb {R}}^n)$ such that

$$\begin{aligned} \Vert u\Vert _{H^{s}(\mathbb {R}^{n})}:= \left\| \langle D\rangle ^s u \right\| _{L^2({\mathbb {R}}^n)}<\infty , \end{aligned}$$

where $\langle D\rangle ^s$ is the Bessel potential operator of order *s* having Fourier symbol $\left( 1+|\xi |^2\right) ^{s/2}$. The fractional Laplacian of order $s\ge 0$ can be defined as a Fourier multiplier

$$\begin{aligned} (-\Delta )^{s} u = \mathcal {F}^{-1}(\left\lvert \xi \right\rvert ^{2s}\widehat{u}(\xi )), \end{aligned}$$

for $u \in \mathscr {S}^{\prime }({\mathbb {R}}^n)$ whenever the right hand side of the above identity is well-defined. In addition, it is also known that for $s\ge 0$, an equivalent norm on $H^s({\mathbb {R}}^n)$ is given by

2.1 $$\begin{aligned} \Vert u\Vert _{H^s({\mathbb {R}}^n)}^*= \Vert u\Vert _{L^{2}(\mathbb {R}^{n})}+\Vert (-\Delta )^{s/2}u \Vert _{L^{2}(\mathbb {R}^{n})}, \end{aligned}$$

and the fractional Laplacian $(-\Delta )^{s}:H^{t}({\mathbb {R}}^n) \rightarrow H^{t-2s}({\mathbb {R}}^n)$ is a bounded linear operator for all $s \ge 0$ and $t \in {\mathbb {R}}$.

The above heuristically introduced UCP reads more formally as follows:

#### Proposition 2.1

(UCP for fractional Laplacians) Let $s> 0$ be a non-integer and $t\in {\mathbb {R}}$. If $u\in H^t({\mathbb {R}}^n)$ satisfies $u=(-\Delta )^s u=0$ in a nonempty open subset $V\subset {\mathbb {R}}^n$, then $u\equiv 0$ in ${\mathbb {R}}^n$.

The preceding proposition was first shown in [11, Theorem 1.2] for the range $s\in (0,1)$, in which case the fractional Laplacian $(-\Delta )^s$ can be equivalently computed as the singular integral

$$ (-\Delta )^su(x)=C_{n,s}\text {p.v.}\int _{{\mathbb {R}}^n}\frac{u(x)-u(y)}{|x-y|^{n+2s}}\,dy $$

for sufficiently nice functions *u* and some constant $C_{n,s}>0$. For the higher order case $s>1$, one can apply the standard Laplacian to the equation, then the classical UCP for the Laplacian yields iteratively the desired result.

For the well-posedness theory, we will use the following Poincaré inequality.

#### Proposition 2.2

(Poincaré inequality (cf. [32, Lemma 5.4])) Let $\Omega \subset {\mathbb {R}}^n$ be a bounded domain. For any $s\ge 0$, there exists $C>0$ such that

$$\begin{aligned} \Vert u\Vert _{L^2(\Omega )}\le C \Vert (-\Delta )^{s/2}u \Vert _{L^2({\mathbb {R}}^n)} \end{aligned}$$

for all $$u\in C_c^{\infty }(\Omega )$$.

Next we introduce some local variants of the above fractional Sobolev spaces. If $\Omega \subset {\mathbb {R}}^n$ is an open set, $F\subset {\mathbb {R}}^n$ a closed set and $s\in \mathbb {R}$, then we set

$$\begin{aligned} H^{s}(\Omega )&:=\left\{ u|_{\Omega } ; \, u\in H^{s}(\mathbb {R}^{n})\right\} ,\\ \widetilde{H}^{s}(\Omega )&:=\text {closure of }C_{c}^{\infty }(\Omega )\text { in }H^{s}(\mathbb {R}^{n}),\\ H_F^s&:=\{u\in H^s({\mathbb {R}}^n)\,;\,{{\,\textrm{supp}\,}}(u)\subset F\}. \end{aligned}$$

Meanwhile, $H^{s}(\Omega )$ is a Banach space with respect to the quotient norm

$$ \Vert u\Vert _{H^{s}(\Omega )}:=\inf \left\{ \Vert U\Vert _{H^{s}(\mathbb {R}^{n})} ; \, U\in H^{s}(\mathbb {R}^{n}) \text{ and } U|_{\Omega }=u\right\} . $$

Hence, using the fact that (2.1) is an equivalent norm on $\widetilde{H}^s(\Omega )$, Propositions 2.2 and the density of $C_c^{\infty }(\Omega )$ in $\widetilde{H}^s(\Omega )$, we have:

#### Lemma 2.3

Let $\Omega \subset {\mathbb {R}}^n$ be a bounded domain and $s\ge 0$. Then an equivalent norm on $\widetilde{H}^s(\Omega )$ is given by

$$\begin{aligned} \Vert u\Vert _{\widetilde{H}^s(\Omega )}=\Vert (-\Delta )^{s/2}u\Vert _{L^2({\mathbb {R}}^n)}. \end{aligned}$$

The observation of Lemma 2.3 will be of constant use in the well-posedness theory below.

### Bochner spaces

Next, we introduce some standard function spaces for time-dependent PDEs adapted to the nonlocal setting considered in this article. Let *X* be a Banach space and $(a,b)\subset {\mathbb {R}}$. Then we let $C^k([a,b];X)$, $L^p(a,b;X)$ ($k\in {\mathbb {N}},1\le p\le \infty$) stand for the space of $k-$times continuously differentiable functions and the space of measurable functions $u:(a,b)\rightarrow X$ such that $t\mapsto \Vert u(t)\Vert _X\in L^p([a,b])$. These spaces carry the norms

2.2 $$\begin{aligned} \begin{aligned} \Vert u\Vert _{L^p(a,b\,;X)}&:= \left( \int _a^b\Vert u(t)\Vert _{X}^p\,dt\right) ^{1/p}<\infty ,\\ \Vert u\Vert _{C^k([a,b];X)}&:= \sup _{0\le \ell \le k}\Vert \partial _t^{\ell }u\Vert _{L^{\infty }([a,b];X)} \end{aligned} \end{aligned}$$

with the usual modifications in the case $p=\infty$.

Additionally, whenever $u\in L^1_{{{\,\textrm{loc}\,}}}(a,b;X)$ with *X* being a space of functions over a subset of some euclidean space, such as $L^2(\Omega )$ or $H^s({\mathbb {R}}^n)$, then *u* is identified with a function *u*(*x*, *t*) and *u*(*t*) denotes the function $x\mapsto u(x,t)$ for almost all *t*. This is justified by the fact, that any $u\in L^q(a,b;L^p(\Omega ))$ with $1\le q,p<\infty$ can be seen as a measurable function $u:\Omega \times (a,b)\rightarrow {\mathbb {R}}$ such that the norm $\Vert u\Vert _{L^q(a,b;L^p(\Omega ))}$, as defined in (2.2), is finite. In particular, one has $L^p(0,T;L^p(\Omega ))=L^p(\Omega _T)$ for $1\le p<\infty$. Clearly, a similar statement holds for the spaces $L^q(a,b;H^{s}({\mathbb {R}}^n))$ and their local versions. If no confusion arises we also denote $L^p(0,T;X)$ by $L^p(X)$ and $L^q(0,T;L^p(\Omega ))$ by $L^qL^p$.

Furthermore, the distributional derivative $\frac{du}{dt}\in \mathscr {D}^{\prime }((a,b);X)$ is identified with the derivative $\partial _tu\in \mathscr {D}^{\prime }(\Omega \times (a,b))$ as long as it is well-defined. Here $\mathscr {D}^{\prime }((a,b);X)$ stands for all continuous linear operators from $C_c^{\infty }((a,b))$ to *X*.

### Abstract framework for solving some 2nd order in time PDEs

We collect here preliminary material to solve some classes of second order PDEs and for a more comprehensive presentation the interested reader can consult [5, Chapter XVIII].

#### Definition 2.4

We say that a tuple (*V*, *H*, *a*, *b*, *c*) consisting of two complex Hilbert spaces *V*, *H*, two families of sesquilinear forms *a*, *b* over *V* and one family of sesquilinear forms *c* over *H* satisfy the *usual conditions* if they have the following properties: The spaces *V* and *H* are such that

2.3 $$\begin{aligned} V\hookrightarrow H\hookrightarrow V' \end{aligned}$$

and the inclusions are dense.^3^ The triple (*a*, *b*, *c*) fulfill the following assumptions:

1. $a(t;\cdot ,\cdot )$, $t\in [0,T]$, is a family of sesquilinear forms over *V*, which can be decomposed as
  $$\begin{aligned} a=a_0+a_1. \end{aligned}$$
  The principal part $a_0$ is required to satisfy
  1. $a_0(t;u,v)\in C^1([0,T])$ for all $u,v\in V$ such that the sesquilinear forms $a_0,\partial _t a_0$ are continuous over *V*,
  2. $$a_0$$ is Hermitian (i.e. $$a_0(t;u,v)=\overline{a_0(t;v,u)}$$),
  3. $a_0$ is coercive over *V* with respect to *H* in the sense that
    $$\begin{aligned} a_0(t;u,u)\ge \alpha \Vert u\Vert _V^2-\lambda \Vert u\Vert _H^2 \end{aligned}$$
    for some $\alpha >0,\lambda \in {\mathbb {R}}$ and all $u\in V$, $t\in [0,T]$.
  The lower order part $a_1$ is assumed to satisfy
  (A4) $$a_1(t;u,v)\in C([0,T])$$ for all $$u,v\in V$$,
  (A5) $|a_1(t;u,v)|\le C\Vert u\Vert _V\Vert v\Vert _H$ for all $u,v\in V$ and $t\in [0,T]$.
2. $b(t;\cdot ,\cdot )$, $t\in [0,T]$, is a family of sesquilinear forms over *V*, which can be decomposed as
  $$\begin{aligned} b=b_0+b_1. \end{aligned}$$
  1. $b_0$ is a continuous sesquilinear form over *V*, Hermitian and coercive in the sense
    $$\begin{aligned} b_0(t;u,u)\ge \mu \Vert u\Vert _V^2 \end{aligned}$$
    for some $\mu >0$ and $t\in [0,T],v\in V$,
  2. $b_1$ is a sesquilinear form satisfying
    $$\begin{aligned} |b_1(t;u,v)|\le C\Vert u\Vert _V\Vert v\Vert _H \end{aligned}$$
    for all $t\in [0,T]$ and $u,v\in V$,
  3. $b_j(t;u,v)\in C([0,T])$ for all $u,v\in V$ and $j=0,1$.
3. $c(t;\cdot ,\cdot )$, $t\in [0,T]$, is a family of sesquilinear forms over *H*, which can be written as
  $$\begin{aligned} c(t;u,v)=\langle C(t)u,v\rangle _H \end{aligned}$$
  for $t\in [0,T]$ and $u,v\in H$. Here *C*(*t*), $t\in [0,T]$, is a family of linear bounded operators on *H* to itself such that
  1. *C*(*t*) is Hermitian and coercive over *H*, that is
    $$\begin{aligned} \langle C(t)u,u\rangle _H\ge \gamma \Vert u\Vert ^2_H \end{aligned}$$
    for some $\gamma >0$ and all $u\in H$, $t\in [0,T]$,
  2. $$\langle C(t)u,v\rangle _H\in C^1([0,T])$$ for all $$u,v\in H$$.

#### Example 2.5

Let $\Omega \subset {\mathbb {R}}^n$ be any bounded Lipschitz domain. Then the separable Hilbert spaces $V=\widetilde{H}^s(\Omega )$ and $H=L^2(\Omega )$^4^ satisfy (2.3) and the inclusions are dense. This is a direct consequence of the Sobolev embedding, the assumption that $\Omega$ is a bounded Lipschitz domain and the fact that $u\in \widetilde{H}^s(\Omega )$ implies $u=0$ a.e. in $\Omega ^c$.

#### Example 2.6

Let *V*, *H* be as in Example 2.5. Then we define the principle part sesquilinear form $a_0:V\times V\rightarrow {\mathbb {C}}$

$$\begin{aligned} a_0(u,v)=\langle (-\Delta )^{s/2} u,(-\Delta )^{s/2} v\rangle _{L^2({\mathbb {R}}^n)} \end{aligned}$$

for all $u,v\in V$. This form clearly satisfies (A1)-(A2) (see Lemma 2.3). The condition (A3) is a consequence of the Poincaré inequality (Proposition 2.2). More precisely, there holds

2.4 $$\begin{aligned} a_0(u,u)\ge c\Vert u\Vert _V^2. \end{aligned}$$

Hence, by the properties of $a_0$, particularly (2.4), we can choose $b_0=a_0$ and the properties (B1) and (B3) are fulfilled. Moreover, we set $b_1=0$ and $C(t)=\text {id}_{L^2(\Omega )}$. Finally, we assume that $q\in L^1_{loc}(\Omega _T)$ is any function such that the induced sesquilinear forms $a_1(t;\cdot ,\cdot ):V\times V\rightarrow {\mathbb {C}}$, $t\in [0,T]$, given by

$$\begin{aligned} a_1(t;u,v)=\langle q(t)u,v\rangle _{L^2(\Omega )} \end{aligned}$$

are well-defined and satisfy (A4), (A5). Hence, the tuple (*V*, *H*, *a*, *b*, *c*) satisfies the usual conditions in the sense of Defintion 2.4.

Next, we introduce several function spaces used throughout this article.

#### Definition 2.7

Let $T>0$. Suppose that we have given Hilbert spaces *V*, *H* satisfying the conditions in Definition 2.4 and a family of bounded linear operators $C(t)\in L(H)$, $t\in [0,T]$, such that the related sesquilinear forms

$$ c(t;u,v)=\langle C(t)u,v\rangle _H $$

fulfill the property (S3) of Definition 2.4.

1. Then we set
  $$\begin{aligned} W_c(0,T;V)=\{v\in L^2(0,T;V)\,;\,\frac{d}{dt}(Cv)\in L^2(0,T;V')\}, \end{aligned}$$
  which carries the norm
  $$\begin{aligned} \Vert v\Vert _{W_c(0,T;V)}=\left( \Vert v\Vert _{L^2(0,T;V)}^2+\Vert \frac{d}{dt}Cv\Vert _{L^2(0,T;V')}^2\right) ^{1/2}. \end{aligned}$$
2. Furthermore, we define
  $$\begin{aligned} \widetilde{W}_c(0,T;V)=\{u\in L^2(0,T;V)\,;\,\partial _t u\in W_c(0,T;V)\} \end{aligned}$$
  and equip it with the norm
  $$\begin{aligned} \Vert u\Vert _{\widetilde{W}_c(0,T;V)}=\left( \Vert u\Vert _{L^2(0,T;V)}^2+\Vert \partial _t u\Vert _{W_c(0,T;V)}^2\right) ^{1/2} \end{aligned}$$

#### Remark 2.8

Clearly both space $W_c(0,T;V)$ and $\widetilde{W}_c(0,T;V)$ are Hilbert spaces. Moreover, if $C(t)=\text {id}_H$, then we drop the subscript *c*.

The next lemma collects a few properties of these spaces (see [5, Chapter XVIII, §5]).

#### Lemma 2.9

Let $T>0$. Suppose that we have given Hilbert spaces *V*, *H* satisfying the conditions in Definition 2.4 and a family of bounded linear operators $C(t)\in L(H)$, $t\in [0,T]$, such that the related sesquilinear forms

$$ c(t;u,v)=\langle C(t)u,v\rangle _H $$

fulfill the property (S3) of Definition 2.4.

(i) One has the embeddings
  2.5 $$\begin{aligned} \widetilde{W}_c(0,T;V)\hookrightarrow C([0,T];V)\text { and }W_c(0,T;V)\hookrightarrow C([0,T];H). \end{aligned}$$
(ii) The space $C_c^{\infty }([0,T];V)$ is dense in $W_c(0,T;V)$ and in $\widetilde{W}_c(0,T;V)$.

Now, we can formulate the abstract forward problem.

#### Problem 2.10

Suppose we have given a tuple (*V*, *H*, *a*, *b*, *c*) satisfying the usual conditions. Does there exist for all functions $u_0\in V$, $u_1\in H$ and $f\in L^2(0,T;V')$ a unique function $u\in \widetilde{W}_c(0,T;V)$ satisfying

2.6 $$\begin{aligned} \frac{d}{dt}\,c(\cdot ;\partial _t u,v)+b(\cdot ;\partial _t u,v)+a(\cdot ;u,v)=\langle f,v\rangle _{V'\times V} \end{aligned}$$

for all $v\in V$ in the sense of $\mathscr {D}^{\prime }((0,T))$ and

2.7 $$\begin{aligned} u(0)=u_0\text { in }V\quad \text { and }\quad \partial _t u(0)=u_1\text { in }H. \end{aligned}$$

#### Remark 2.11

Note that $u\in \widetilde{W}_c(0,T;V)$ guarantees that all terms in (2.6) are well-defined and the embeddings (2.5) ensure that (2.7) makes sense.

Now, we can state the main well-posedness result on abstract second order in time PDEs, which we will use later on.

#### Theorem 2.12

(Well-posedness abstract PDEs) Let $T>0$. Assume (*V*, *H*, *a*, *b*, *c*) consisting of two complex Hilbert spaces *V*, *H*, two sesquilinear forms *a*, *b* over *V* and a sesquilinear form over *H* satisfy the usual conditions. Then for any $u_0\in V$, $u_1\in H$ and $f\in L^2(0,T;V')$, the Problem 2.10 has a unique solution $u\in \widetilde{W}_c(0,T;V)$.

#### Proof

This result is a direct consequence of [5, Chapter XVIII, §5, Theorem 1, Remark 4]. $\square$

## Well-posedness theory of viscous wave equations

In this section, we study the well-posedness theory of the viscous wave equation with linear and nonlinear perturbations.

### Viscous wave equation with linear perturbations

Let us start by stating the well-posedness result in the linear case.

#### Theorem 3.1

Let $\Omega \subset {\mathbb {R}}^n$ be a bounded Lipschitz domain, $T>0$ and $s>0$. Suppose that the (real valued) function $q\in L^1_{loc}(\Omega _T)$ has the following properties:

(i) $q\in L^{\infty }(0,T;L^p(\Omega ))$ for some $1\le p<\infty$ satisfying
  $$\begin{aligned} {\left\{ \begin{array}{ll} n/s\le p\le \infty , & \, \text {if }\, 2s< n,\\ 2<p\le \infty , & \, \text {if }\, 2s= n,\\ 2\le p\le \infty , & \, \text {if }\, 2s > n, \end{array}\right. } \end{aligned}$$
(ii) $t\mapsto \int _{\Omega }q(x,t)\varphi (x)\,dx\in C([0,T])$ for any $\varphi \in C_c^{\infty }(\Omega )$.

Then for any pair $(u_0, u_1)\in \widetilde{H}^s(\Omega )\times L^2(\Omega )$ and $h\in L^2(0,T;H^{-s}(\Omega ))$ there exists a unique solution $u\in \widetilde{W}(0,T;\widetilde{H}^s(\Omega ))$ of

3.1 $$\begin{aligned} {\left\{ \begin{array}{ll} \left( \partial _t^2 +(-\Delta )^s\partial _t +(-\Delta )^s+q\right) u=h & \text { in }\Omega _T\\ u=0 & \text { in }(\Omega _e)_T,\\ u(0)=u_0,\quad \partial _t u(0)=u_1 & \text { in }\Omega . \end{array}\right. } \end{aligned}$$

Moreover, *u* satisfies the following energy identity^5^

3.2 $$\begin{aligned} \begin{aligned}&\Vert \partial _t u(t) \Vert _{L^2(\Omega )}^2+ \Vert (-\Delta )^{s/2}u(t)\Vert _{L^2({\mathbb {R}}^n)}^2+ 2\Vert (-\Delta )^{s/2}\partial _tu\Vert _{L^2({\mathbb {R}}^n_t)}^2\\&\quad =\Vert u_1 \Vert _{L^2(\Omega )}^2+\Vert (-\Delta )^{s/2}u_0 \Vert _{L^2({\mathbb {R}}^n)}^2+2\int _0^t\langle h(\tau ),\partial _t u (\tau )\rangle \,d\tau -2\langle q u,\partial _t u \rangle _{L^2(\Omega _t)} \end{aligned} \end{aligned}$$

for all $t\in [0,T]$. Moreover, if $(u_{0,j},u_{1,j})\in \widetilde{H}^s(\Omega )\times L^2(\Omega )$, $h_j\in L^2(0,T;H^{-s}(\Omega ))$ and $u_j\in \widetilde{W}(0,T;\widetilde{H}^s(\Omega ))$ denote the related unique solution to (3.1) for $j=1,2$, then the following continuity estimate holds

3.3 $$\begin{aligned} \begin{aligned}&\Vert u_1-u_2\Vert _{L^{\infty }(0,T;\widetilde{H}^s(\Omega ))}+\Vert \partial _t u_1-\partial _t u_2\Vert _{L^{\infty }(0,T;L^2(\Omega ))}+\Vert \partial _t u_1-\partial _t u_2\Vert _{L^{2}(0,T;\widetilde{H}^s(\Omega ))}\\&\quad \le C(\Vert u_{0,1}-u_{0,2}\Vert _{\widetilde{H}^s(\Omega )}+\Vert u_{1,1}-u_{1,2}\Vert _{L^2(\Omega )}+\Vert h_1-h_2\Vert _{L^2(0,T;H^{-s}(\Omega ))}) \end{aligned} \end{aligned}$$

for some $C>0$ depending on $T>0$.

#### Proof

For the time being assume $\widetilde{H}^s(\Omega )$ and $L^2(\Omega )$ consist of complex functions. We claim that we are in the setting of Example 2.6. To this end, we only need to verify that the sesquilinear form

$$\begin{aligned} a_1(t;u,v)=\langle q(t)u,v\rangle _{L^2(\Omega )} \end{aligned}$$

for $u,v\in \widetilde{H}^s(\Omega )$ satisfies (A4) and (A5). If we can show the estimate

3.4 $$\begin{aligned} \begin{aligned} \left| \langle qu,v\rangle _{L^2(\Omega )} \right|&\le C\Vert q(t)\Vert _{L^p(\Omega )}\Vert u\Vert _{\widetilde{H}^s(\Omega )}\Vert v\Vert _{L^2(\Omega )}\\&\le C\Vert q\Vert _{L^{\infty }(0,T;L^p(\Omega ))}\Vert u\Vert _{\widetilde{H}^s(\Omega )}\Vert v\Vert _{L^2(\Omega )} \end{aligned} \end{aligned}$$

for some $C>0$ independent of $t\in [0,T]$, then (A5) follows. The case $p=\infty$ is clear. In the case $\frac{n}{s}\le p<\infty$ with $2s< n$ one can use Hölder’s inequality with

$$ \frac{1}{2}=\frac{n-2s}{2n}+\frac{s}{n}, $$

$L^{r_2}(\Omega )\hookrightarrow L^{r_1}(\Omega )$ for $r_1\le r_2$ as $\Omega \subset {\mathbb {R}}^n$ is bounded and Sobolev’s inequality to obtain

3.5 $$\begin{aligned} \begin{aligned} \left| \langle qu,v\rangle _{L^2(\Omega )} \right|&\le \Vert qu\Vert _{L^2(\Omega )}\Vert v\Vert _{L^2(\Omega )}\\&\le \Vert q\Vert _{L^{n/s}(\Omega )}\Vert u\Vert _{L^{\frac{2n}{n-2s}}(\Omega )}\Vert v\Vert _{L^2(\Omega )}\\&\le C\Vert q\Vert _{L^{n/s}(\Omega )}\Vert u\Vert _{L^{\frac{2n}{n-2s}}(\Omega )}\Vert v\Vert _{L^2(\Omega )}\\&\le C\Vert q\Vert _{L^{p}(\Omega )}\Vert (-\Delta )^{s/2} u \Vert _{L^2({\mathbb {R}}^n)}\Vert v\Vert _{L^2(\Omega )} \end{aligned} \end{aligned}$$

for all $u,v\in \widetilde{H}^s(\Omega )$. In the case $2s>n$ one can use the embedding $H^s({\mathbb {R}}^n)\hookrightarrow L^{\infty }({\mathbb {R}}^n)$ together with Lemma 2.3 and the boundedness of $\Omega$ to see that the estimate (3.5) holds. In the case $n=2s$ one can use the boundedness of the embedding $\widetilde{H}^s(\Omega )\hookrightarrow L^{\overline{p}}(\Omega )$ for all $2\le \overline{p}<\infty$, Hölder’s inequality and the boundedness of $\Omega$ to get the estimate (3.5). In fact, the aforementioned embedding in the critical case follows by [28] and the Poincaré inequality.

Next, we show that the condition (ii) implies (A4). For this purpose, let us define for any $t\in [0,T]$ the function

$$ \Phi _{\varphi }(t)=\int _{\Omega }q(x,t)\varphi (x)\,dx $$

for any $\varphi \in L^1_{loc}(\Omega )$ such that the integral is well-defined. Suppose $u_j,v_j\in \widetilde{H}^s(\Omega )$, $j=1,2$, are given. Then the estimate (3.5) implies

3.6 $$\begin{aligned} \begin{aligned}&|\Phi _{u_1\overline{v_1}}(t)-\Phi _{u_2\overline{v_2}}(t)|\\&\quad \le |\Phi _{(u_1-u_2)\overline{(v_1-v_2)}}(t)|+|\Phi _{(u_1-u_2)\overline{v_2}}(t)|+|\Phi _{u_2\overline{(v_1-v_2)}}(t)|\\&\quad \le C(\Vert u_1-u_2\Vert _{\widetilde{H}^s(\Omega )}\Vert v_1-v_2\Vert _{L^2(\Omega )}+\Vert u_1-u_2\Vert _{\widetilde{H}^s(\Omega )}\Vert v_2\Vert _{L^2(\Omega )}+\Vert u_2\Vert _{\widetilde{H}^s(\Omega )}) \end{aligned} \end{aligned}$$

for all $t\in [0,T]$. Now, for fixed $u,v\in \widetilde{H}^s(\Omega )$ there exist sequences $u_k,v_k\in C_c^{\infty }(\Omega )$ such that

$$ u_k\rightarrow u\text { and }v_k\rightarrow v\text { in }\widetilde{H}^s(\Omega ) $$

as $k\rightarrow \infty$. Then the estimate (3.6) shows that

$$ \Phi _{\varphi _k}(t)\rightarrow \Phi _{u\overline{v}}(t)\text { as }k\rightarrow \infty $$

uniformly in $t\in [0,T]$, where $\varphi _k=u_k\overline{v_k}\in C_c^{\infty }(\Omega )$. Hence, for any $\varepsilon >0$ there exists $k_0\in {\mathbb {N}}$ such that

$$ \Vert \Phi _{\varphi _k}-\Phi _{u\overline{v}}\Vert _{L^{\infty }([0,T])}<\varepsilon /3 $$

for all $k\ge k_0$. Now, let us fix such a $k\ge k_0$. On the other hand, for any $k\in {\mathbb {N}}$ we know by (ii) that $\Phi _{\varphi _k}\in C([0,T])$ and thus for given $k\ge k_0$ we can choose $\delta =\delta (k)>0$ such that if $t_1,t_2\in [0,T]$ with $|t_1-t_2|<\delta$, we have

$$ |\Phi _{\varphi _k}(t_1)-\Phi _{\varphi _k}(t_2)|<\varepsilon /3. $$

Thus, we get

$$ \begin{aligned}&|\Phi _{u\overline{v}}(t_1)-\Phi _{u\overline{v}}(t_2)|\\&\quad \le |\Phi _{u\overline{v}}(t_1)-\Phi _{\varphi _k}(t_1)|+|\Phi _{u\overline{v}}(t_2)-\Phi _{\varphi _k}(t_2)|+|\Phi _{\varphi _k}(t_1)-\Phi _{\varphi _k}(t_2)|<\varepsilon \end{aligned} $$

for all $t_1,t_2\in [0,T]$ such that $|t_1-t_2|<\delta$. Hence, we have $\Phi _{u\overline{v}}\in C([0,T])$ and so the sesquilinear form $a_1$ satisfies (A4) as well.

Therefore, if we extend $h\in L^2(0,T;H^{-s}(\Omega ))=L^2(0,T;(\widetilde{H}^s(\Omega ))^*)$ to its unique antilinear functional $H\in L^2(0,T;(\widetilde{H}^s(\Omega ))')$, we may deduce from Theorem 2.12 the existence of a unique solution $\widetilde{u}\in \widetilde{W}(0,T;\widetilde{H}^s(\Omega ))$ of (2.6), where (*V*, *H*, *a*, *b*, *c*) are as above. Since *q* is real valued, one easily deduces the existence of a unique real valued solution $u\in \widetilde{W}(0,T;\widetilde{H}^s(\Omega ))$ of (3.1).

The energy identity (3.2) and the continuity estimate (3.6) are direct consequences of [5, Chapter XVIII, §5, Equations (5.81)-(5.82) and Remark 4] and the fact that all appearing functions are real valued. $\square$

Next, we introduce several function spaces, which are used below. First, we define

$$\begin{aligned} \widetilde{W}_*(0,T;H^s({\mathbb {R}}^n))=\{v\in \widetilde{W}(0,T;H^s({\mathbb {R}}^n))\,;\,v(0)\in \widetilde{H}^s(\Omega ),\,\partial _t v(0)\in L^2(\Omega )\}. \end{aligned}$$

By Lemma 2.9 it follows that $\widetilde{W}_*(0,T;H^s({\mathbb {R}}^n))\subset \widetilde{W}(0,T;H^s({\mathbb {R}}^n))$ is a closed subspace and thus again a Hilbert space. Additionally, we introduce the Banach space

$$\begin{aligned} \widetilde{W}_{ext}(0,T;\widetilde{H}^s(\Omega ))=\widetilde{W}(0,T;\widetilde{H}^s(\Omega ))+\widetilde{W}_*(0,T;H^s({\mathbb {R}}^n))\subset H^1(0,T;H^s({\mathbb {R}}^n)), \end{aligned}$$

which is as usual endowed with the norm

$$\begin{aligned} \Vert u\Vert _{\widetilde{W}_{ext}(0,T;\widetilde{H}^s(\Omega ))}=\inf (\Vert v\Vert _{\widetilde{W}(0,T;\widetilde{H}^s(\Omega ))}+\Vert \varphi \Vert _{\widetilde{W}(0,T;H^s({\mathbb {R}}^n))}), \end{aligned}$$

where the infimum is taken over all $v\in \widetilde{W}(0,T;\widetilde{H}^s(\Omega ))$ and $\varphi \in \widetilde{W}_*(0,T;H^s({\mathbb {R}}^n))$ such that $u=v+\varphi$. Later $\widetilde{W}_{ext}(0,T;\widetilde{H}^s(\Omega ))$ will play the role of the solution space to problems with nonzero exterior conditions $\varphi$.

The last space we need is

$$ \widetilde{W}^s_\text {rest}((\Omega _e)_T)=\{v|_{(\Omega _e)_T}\,;\,v\in \widetilde{W}_*(0,T;H^s({\mathbb {R}}^n))\}, $$

which is a Banach space when endowed with the corresponding quotient norm.

#### Corollary 3.2

Let $\Omega \subset {\mathbb {R}}^n$ be a bounded Lipschitz domain, $T>0$ and $s>0$. Suppose that the (real valued) function $q\in L^1_{loc}(\Omega _T)$ satisfies the conditions in Theorem 3.1.

(i) For any pair $(u_0, u_1)\in \widetilde{H}^s(\Omega )\times L^2(\Omega )$, $h\in L^2(0,T;H^{-s}(\Omega ))$ and $\Phi \in \widetilde{W}_*(0,T;\widetilde{H}^s({\mathbb {R}}^n))$, there exists a unique solution $u\in \widetilde{W}_{ext}(0,T;\widetilde{H}^s(\Omega ))$ of
  3.7 $$\begin{aligned} {\left\{ \begin{array}{ll} \left( \partial _t^2 +(-\Delta )^s\partial _t +(-\Delta )^s+q\right) u=h & \text { in }\Omega _T\\ u=\Phi & \text { in }(\Omega _e)_T,\\ u(0)=u_0,\quad \partial _t u(0)=u_1 & \text { in }\Omega . \end{array}\right. } \end{aligned}$$
  This means
  (I) for all $v\in \widetilde{H}^s(\Omega )$ one has
    $$\begin{aligned} \begin{aligned}&\frac{d}{dt}\,\langle \partial _t u,v\rangle _{L^2(\Omega )}+\langle (-\Delta )^{s/2}\partial _t u,(-\Delta )^{s/2}v\rangle _{L^2({\mathbb {R}}^n)}\\&+\langle (-\Delta )^{s/2}u,(-\Delta )^{s/2}v\rangle _{L^2({\mathbb {R}}^n)}+\langle q u,v\rangle _{L^2(\Omega )}=\langle h,v\rangle \end{aligned} \end{aligned}$$
    in the sense of $\mathscr {D}^{\prime }((0,T))$,
  (II) $$u=\Phi $$ in $$(\Omega _e)_T$$,
  (III) $u(0)=u_0$ in $\widetilde{H}^s(\Omega )$ and $\partial _t u(0)=u_1$ in $L^2(\Omega )$.
(ii) If $\varphi \in \widetilde{W}^s_{rest}((\Omega _e)_T)$ and $\Phi _1,\Phi _2$ are any two representations of $\varphi$ with unique solutions $u_1$ and $u_2$ of (3.7) with $\Phi =\Phi _1$ and $\Phi =\Phi _2$, respectively, then there holds $u_1=u_2$. In particular, for any $\varphi \in \widetilde{W}^s_{rest}((\Omega _e)_T)$ we have a unique solution *u* of (3.7).

#### Proof

(i): We first observe that if $u\in \widetilde{W}_{ext}(0,T;\widetilde{H}^s(\Omega ))$ and $\Phi \in \widetilde{W}_*(0,T;H^s({\mathbb {R}}^n))$, then one has $u=\Phi$ in $(\Omega _e)_T$ if and only if

3.8 $$\begin{aligned} u-\Phi \in \widetilde{W}(0,T;\widetilde{H}^s(\Omega )). \end{aligned}$$

In fact, if $u=v+\psi$ with $v\in \widetilde{W}(0,T;\widetilde{H}^s(\Omega ))$ and $\psi \in \widetilde{W}_*(0,T;H^s({\mathbb {R}}^n))$, then one has $u=\Phi$ in $(\Omega _e)_T$ if and only if $\psi =\Phi$ in $(\Omega _e)_T$. As $\Omega$ has a Lipschitz continuous boundary, one knows that functions in $\widetilde{H}^s(\Omega )$ coincide with $H^s({\mathbb {R}}^n)$ functions vanishing a.e. in $\Omega ^c$. Thus, we have $\psi -\Phi \in \widetilde{W}(0,T;\widetilde{H}^s(\Omega ))$ and thus $u-\Phi \in \widetilde{W}(0,T;\widetilde{H}^s(\Omega ))$. On the other hand, the condition (3.8) clearly implies $u=\Phi$ in $(\Omega _e)_T$.

By the regularity assumptions of the involved functions and the above equivalent reformulation of the exterior condition, this means nothing else than that $v=u-\Phi \in \widetilde{W}(0,T;\widetilde{H}^s(\Omega ))$ solves (3.1), where the right hand side is given by

3.9 $$\begin{aligned} h-(\partial _t^2\Phi +(-\Delta )^s\partial _t \Phi +(-\Delta )^s\Phi +q\Phi )\in L^2(0,T;H^{-s}(\Omega )) \end{aligned}$$

and the initial conditions by $u_0-\Phi (0)$, $u_1-\partial _t\Phi (0)$. To see the regularity condition in (3.9) recall the estimate (3.4) from the proof of Theorem 3.1. As this problem is well-posed the same holds for problem (3.7).

(ii): One easily sees that $u_1-u_2$ belongs to $\widetilde{W}(0,T;\widetilde{H}^s(\Omega ))$ and is the unique solution of

$$\begin{aligned} {\left\{ \begin{array}{ll} \left( \partial _t^2 +(-\Delta )^s\partial _t +(-\Delta )^s+q\right) u=0 & \text { in }\Omega _T\\ u=0 & \text { in }(\Omega _e)_T,\\ u(0)=0,\quad \partial _t u(0)=0 & \text { in }\Omega . \end{array}\right. } \end{aligned}$$

From Theorem 3.1 we deduce $u_1=u_2$. Hence, we can conclude the proof. $\square$

### Viscous wave equation with nonlinear perturbations

In this section we establish the well-posedness of the viscous wave equation with nonlinear perturbations. To this end we first discuss in Sect.  3.2.1 the continuity and differentiablity of the Nemytskii operator *f*(*u*) under certain assumptions on *f* (see Assumption 3.4). Then in Sect. 3.2.2 we invoke the implicit function theorem to get the desired well-posedness result and see that the solution map $S(\varphi )$ depends differentiabily on the exterior condition for small $\varphi$.

#### Differentiability of nonlinear perturbations

Next, we move on to the nonlinear problem. For this purpose let us specify a class of nonlinearities *f* containing the one considered in the PDE (1.7). We start by recalling the notion of a Carathéodory function.

##### Definition 3.3

Let $U\subset {\mathbb {R}}^n$ be an open set. We say that $f:U\times {\mathbb {R}}\rightarrow {\mathbb {R}}$ is a *Carathódory function*, if it has the following properties:

(i) $\tau \mapsto f(x,\tau )$ is continuous for a.e. $x\in U$,
(ii) $x\mapsto f(x,\tau )$ is measurable for all $\tau \in {\mathbb {R}}$.

##### Assumption 3.4

Let $f:\Omega \times {\mathbb {R}}\rightarrow {\mathbb {R}}$ a Carathéodory function satisfying the following conditions:

(i) *f* has partial derivative $\partial _{\tau }f$, which is a Carathéodory function,
(ii) and there exists $a\in L^p(\Omega )$ such that
  3.10 $$\begin{aligned} \left| \partial _\tau f(x,\tau )\right| \lesssim a(x)+|\tau |^r \end{aligned}$$
  for all $\tau \in {\mathbb {R}}$ and a.e. $x\in \Omega$. Here the exponents *p* and *r* satisfy the restrictions
  $$\begin{aligned} {\left\{ \begin{array}{ll} n/s\le p\le \infty , & \, \text {if }\, 2s< n,\\ 2<p\le \infty , & \, \text {if }\, 2s= n,\\ 2\le p\le \infty , & \, \text {if }\, 2s > n, \end{array}\right. } \end{aligned}$$
  and
  3.11 $$\begin{aligned} {\left\{ \begin{array}{ll} 0\le r<\infty , & \, \text {if }\, 2s \ge n,\\ 0\le r\le \frac{2s}{n-2s}, & \, \text {if }\, 2s< n, \end{array}\right. } \end{aligned}$$
  respectively.

##### Remark 3.5

An example of a nonlinearity $f$ that satisfies the conditions in Assumption 3.4 is the fractional power–type nonlinearity

$$ f(x,\tau ) = q(x)\,|\tau |^{r}\tau , $$

where $r \ge 0$ satisfies (3.11) and $q \in L^{\infty }(\Omega )$. The regularity conditions are clearly fulfilled. Moreover, a direct computation shows that

$$\begin{aligned} \partial _\tau f(x,\tau ) = (r+1)\, q(x)\, |\tau |^{r}, \end{aligned}$$

and therefore, in this case, we may take $a = 0$ in (3.10).

Next, we state to auxiliary lemmas on the continuity and differentiability of Nemytskii operators.

##### Lemma 3.6

(Continuity of Nemytskii operators) Let $\Omega \Subset {\mathbb {R}}^n$, $T>0$, $1\le q,p<\infty$ and assume that $f:\Omega \times {\mathbb {R}}\rightarrow {\mathbb {R}}$ is a Carathéodory function satisfying

3.12 $$\begin{aligned} |f(x,\tau )|\le a+b|\tau |^{\alpha } \end{aligned}$$

for some constants $a,b\ge 0$ and $0<\alpha \le \min (p,q)$. Then the Nemytskii operator *f*, defined by

$$\begin{aligned} f(u)(x,t):= f(x,u(x,t)) \end{aligned}$$

for all measurable functions $u:\Omega _T\rightarrow {\mathbb {R}}$, maps continuously $L^q(0,T;L^p(\Omega ))$ into $L^{q/\alpha }(0,T;L^{p/\alpha }(\Omega ))$.

##### Proof

The measurability and that the Nemytskii operator *f* is well-defined, are immediate. Thus, we only need to check that it is continuous.

Let $(u_n)_{n\in {\mathbb {N}}}\subset L^qL^p$ such that $u_n\rightarrow u$ in $L^qL^p$ as $n\rightarrow \infty$. By the converse of the dominated convergence theorem, we know that there is a subsequence, still denoted by $(u_n)$, and a function $g\in L^q((0,T))$ such that

- $$u_n(t)\rightarrow u(t)$$ in $$L^p(\Omega )$$ for a.e. *t*,
- $$\Vert u_n(t)\Vert _{L^p(\Omega )}\le g(t)$$ for a.e. *t*.

By [1, Theorem 2.2] we know that *f* is continuous from $L^p(\Omega )$ to $L^{p/\alpha }(\Omega )$ and so taking into account the estimate (3.12) as well as (a), (b), we deduce that

(A) $$f(u_n(t))\rightarrow f(u(t))$$ in $$L^{p/\alpha }(\Omega )$$ for a.e. *t*,
(B) $$\Vert f(u_n(t))\Vert _{L^{p/\alpha }(\Omega )}\le C(1+\Vert u_n(t)\Vert _{L^p(\Omega )}^{\alpha })\le C(1+g(t)^{\alpha })\in L^{q/\alpha }((0,T))$$.

Applying the dominated convergence theorem shows that $f(u_n)\rightarrow f(u)$ in $L^{q/\alpha }L^{p/\alpha }$ as $n\rightarrow \infty$. Since this holds for every subsequence of $(u_n)_{n\in {\mathbb {N}}}$, we see that the whole sequence $(f(u_n))_{n\in {\mathbb {N}}}$ converges to *f*(*u*). Hence, we can conclude the proof. $\square$

##### Lemma 3.7

(Differentiability of Nemytskii operators) Let $\Omega \Subset {\mathbb {R}}^n$ be a domain, $T>0$, $2<p<\infty$, $p-1\le q<\infty$ and assume that $f:\Omega \times {\mathbb {R}}\rightarrow {\mathbb {R}}$ is a Carathéodory function with $f(\cdot ,0)\in L^{\infty }(\Omega )$. Moreover, suppose that *f* has partial derivative $\partial _\tau f$, which is a Carathéodory function and satisfies the estimate

3.13 $$\begin{aligned} \left| \partial _{\tau }f(x,\tau )\right| \le a+b|\tau |^{p-2} \end{aligned}$$

for some constants $a,b\ge 0$. Then the Nemytskii operator *f* is Fréchet differentiable as a map from $L^q(0,T;L^p(\Omega ))$ to $L^{\frac{q}{p-1}}(0,T;L^{\frac{p}{p-1}}(\Omega ))$ with differential

$$\begin{aligned} df(u)h=\partial _\tau f(u)h. \end{aligned}$$

##### Proof

An integration of (3.13) shows that *f* satisfies the growth condition

$$\begin{aligned} |f(x,\tau )|\le c+d|\tau |^{p-1} \end{aligned}$$

for some constants $c,d\ge 0$. By Lemma 3.6 it follows that *f* and $\partial _{\tau }f$ are continuous as mappings

3.14 $$\begin{aligned} \begin{aligned} f:L^q(0,T;L^p(\Omega ))&\rightarrow L^{\frac{q}{p-1}}(0,T;L^{\frac{p}{p-1}}(\Omega ))\\ \partial _{\tau }f:L^q(0,T;L^p(\Omega ))&\rightarrow L^{\frac{q}{p-2}}(0,T;L^{\frac{p}{p-2}}(\Omega )). \end{aligned} \end{aligned}$$

Next, let $u,h\in L^qL^p$ and define

$$ \omega (u,h)=\Vert f(u+h)-f(u)-\partial _\tau f(u) h\Vert _{L^{\frac{q}{p-1}}L^{\frac{p}{p-1}}}. $$

By [1, eq. (2.8)], we know

$$ \omega (u,h)\le \left\| \Vert h\Vert _{L^p}\left\| \int _0^1\left( \partial _\tau f(u+\xi h)-\partial _\tau f(u)\right) \,d\xi \right\| _{L^{\frac{p}{p-2}}}\right\| _{L^{\frac{q}{p-1}}}. $$

Observing that

$$ \frac{p-1}{q}=\frac{1}{q}+\frac{p-2}{q}, $$

we get by Hölder’s and Minkowski’s inequality

$$ \begin{aligned} \omega (u,h)&\le \Vert h\Vert _{L^qL^p}\left\| \int _0^1\left( \partial _\tau f(u+\xi h)-\partial _\tau f(u)\right) d\xi \right\| _{L^{\frac{q}{p-2}}L^{\frac{p}{p-2}}}\\&\le \Vert h\Vert _{L^qL^p}\int _0^1\left\| \partial _\tau f(u+\xi h)-\partial _\tau f(u)\right\| _{L^{\frac{q}{p-2}}L^{\frac{p}{p-2}}}d\xi . \end{aligned} $$

By (3.14) the second factor goes to zero as $h\rightarrow 0$ in $L^qL^p$ and therefore we get $\omega (u,h)=o(\Vert h\Vert _{L^qL^p})$. Hence, *f* is a differentiable map from $L^qL^p$ to $L^{\frac{q}{p-1}}L^{\frac{p}{p-1}}$. $\square$

#### Differentiability of solution map to the nonlinear problem

Now with the tools from the preceding section at our disposal, we can show that the viscous wave equation with nonlinear perturbations is well-posed for small exterior conditions.

##### Theorem 3.8

Let $\Omega \subset {\mathbb {R}}^n$ be a bounded Lipschitz domain, $T>0$ and $s>0$. Suppose that *f* satisfies $f(0)=0$ and Assumption 3.4 with $r>0$ and $a\in L^{\infty }(\Omega )$. Then there exist neighborhoods $U_0\subset \widetilde{W}^s_\text {rest}((\Omega _e)_T)$, $U_1\subset \widetilde{W}_{ext}(0,T;\widetilde{H}^s(\Omega ))$ of the origin with the property that, for any $\varphi \in U_0$, the problem

3.15 $$\begin{aligned} {\left\{ \begin{array}{ll} \partial _t^2u +(-\Delta )^s\partial _t u+(-\Delta )^su+f(u)= 0& \text { in }\Omega _T\\ u=\varphi & \text { in }(\Omega _e)_T,\\ u(0)=0,\quad \partial _t u(0)=0 & \text { in }\Omega \end{array}\right. } \end{aligned}$$

has a unique solution $u\in U_1$ in the sense that:

(i) for all $v\in \widetilde{H}^s(\Omega )$ one has
  $$\begin{aligned} \begin{aligned}&\frac{d}{dt}\,\langle \partial _t u,v\rangle _{L^2(\Omega )}+\langle (-\Delta )^{s/2}\partial _t u,(-\Delta )^{s/2}v\rangle _{L^2({\mathbb {R}}^n)}\\&+\langle (-\Delta )^{s/2}u,(-\Delta )^{s/2}v\rangle _{L^2({\mathbb {R}}^n)}+\langle f(u),v\rangle _{L^2(\Omega )}=0 \end{aligned} \end{aligned}$$
  in the sense of $\mathscr {D}^{\prime }((0,T))$;
(ii) $$u=\varphi $$ in $$(\Omega _e)_T$$;
(iii) $u(0)=0$ in $\widetilde{H}^s(\Omega )$ and $\partial _t u(0)=0$ in $L^2(\Omega )$.

Moreover, the map $U_0\ni \varphi \mapsto S(\varphi )\in U_1$, which assigns to each exterior condition $\varphi \in U_0$ the corresponding unique solution $S(\varphi ):= u$ of (3.15), is $C^1$ in the Fréchet sense.

##### Remark 3.9

Heuristically, the map *S* assigns to each "small" exterior condition $\varphi$ its unique "small" solution $S(\varphi )=u$ and we refer to *S* as the *solution map* associated with the problem (3.15).

##### Proof

Let us start by defining the following Banach spaces

$$\begin{aligned} \begin{aligned} E_0&=\widetilde{W}^s_\text {rest}((\Omega _e)_T),\quad E_1=\widetilde{W}_{ext}(0,T;\widetilde{H}^s(\Omega )),\\ V_0&=\widetilde{H}^s(\Omega ),\quad V_1=L^2(\Omega ),\quad V_2=\widetilde{W}^s_\text {rest}((\Omega _e)_T),\quad V_3=L^{2}(0,T;H^{-s}(\Omega )), \end{aligned} \end{aligned}$$

and define the map $F: E_0\times E_1\rightarrow \prod _{j=0}^3 V_j$ via

$$\begin{aligned} F(\varphi ,u) =\left( u(0), \partial _tu(0),(u-\varphi )|_{(\Omega _e)_T},(\partial _t^2+(-\Delta )^s\partial _t+(-\Delta )^s)u+f(u)\right) . \end{aligned}$$

We now wish to argue that *F* is well-defined. For this purpose we first show that there holds

3.16 $$\begin{aligned} \begin{aligned} \Vert f(\psi )\Vert _{L^2(\Omega _T)} \lesssim \,&(\Vert a\Vert _{L^{\infty }(\Omega )}\Vert \psi \Vert _{L^{\infty }(0,T;H^s({\mathbb {R}}^n))} +\Vert \psi \Vert ^{r+1}_{L^{\infty }(0,T;H^s({\mathbb {R}}^n))}). \end{aligned} \end{aligned}$$

for any $\psi \in C([0,T];H^s({\mathbb {R}}^n))$. First of all by the fundamental theorem of calculus, Assumption 3.4 and $f(0)=0$ we have

$$\begin{aligned} |f(x,s)|\le \left| \int _0^s \partial _{\tau }f(x,\tau )\,d\tau \right| \le C\left( a(x)|s|+|s|^{r+1}\right) , \end{aligned}$$

where $a \in L^{\infty }(\Omega )$ is nonnegative. This ensures that we have

$$ |f(\psi (t))|\le C\left( a|\psi (t)|+|\psi (t)|^{r+1}\right) . $$

Hence, we get

$$\begin{aligned} \Vert f(\psi (t))\Vert _{L^2(\Omega )}\le C\left( \Vert a\psi (t)\Vert _{L^2(\Omega )}+\Vert \psi (t)\Vert _{L^{2(r+1)}(\Omega )}^{r+1}\right) , \end{aligned}$$

for $0\le t\le T$. By the boundedness of *a*, we obtain

$$\begin{aligned} \Vert a\psi (t)\Vert _{L^2(\Omega )}\le \Vert a\Vert _{L^{\infty }(\Omega )}\Vert \psi (t)\Vert _{H^s({\mathbb {R}}^n)}. \end{aligned}$$

Note that the conditions on the exponent *r* yield

$$ {\left\{ \begin{array}{ll} 1\le 1+r<\infty , & \, \text {if }\, 2s\ge n, \\ 1\le 1+r\le \frac{n}{n-2s} & \, \text {if }\, 2s< n. \end{array}\right. } $$

If $2s> n$, then the Sobolev embedding $H^s({\mathbb {R}}^n)\hookrightarrow L^{\infty }({\mathbb {R}}^n)$ yields

$$\begin{aligned} \Vert \psi (t)\Vert _{L^{2(r+1)}(\Omega )}^{r+1}\le C\Vert \psi (t)\Vert _{H^s({\mathbb {R}}^n)}^{r+1}. \end{aligned}$$

In the critical case $2s=n$, we can apply [28] to obtain

$$\begin{aligned} \Vert \psi (t)\Vert _{L^{2(r+1)}(\Omega )}^{r+1}\le C\Vert (-\Delta )^{s/2}\psi (t)\Vert _{L^2({\mathbb {R}}^n)}^r\Vert \psi (t)\Vert _{L^2({\mathbb {R}}^n)}. \end{aligned}$$

In the subcritical case $2s<n$, we apply the Hardy–Littlewood–Sobolev lemma to deduce

$$\begin{aligned} \Vert \psi (t)\Vert _{L^{2(r+1)}(\Omega )}^{r+1}\le C\Vert \psi (t)\Vert _{L^{\frac{2n}{n-2s}}(\Omega )}^{r+1}\le C \Vert (-\Delta )^{s/2}\psi (t) \Vert _{L^2({\mathbb {R}}^n)}^{r+1}. \end{aligned}$$

As $\psi \in C([0,T];H^s({\mathbb {R}}^n))$, we get by the continuity of the fractional Laplacian the desired estimate (3.16).

Now we can show that *F* is well-defined. Since $u\in \widetilde{W}_{ext}(0,T;\widetilde{H}^s(\Omega ))$, we have by definition $u(0)\in \widetilde{H}^s(\Omega )$ and $\partial _t u(0)\in L^2(\Omega )$. Thus, the first two entries of *F* are well-defined. On the other hand as $u=v+\psi \in \widetilde{W}_{ext}(0,T;\widetilde{H}^s(\Omega ))$, we have $(u-\varphi )|_{(\Omega _e)_T}=(\psi -\varphi )|_{(\Omega _e)_T}\in \widetilde{W}^s_\text {rest}((\Omega _e)_T)$. Finally, using the mapping properties of $(-\Delta )^s$, Lemma 2.9 and (3.16), one easily sees that $\partial _t^2 u+(-\Delta )^s\partial _t+(-\Delta )^s u+f(u)\in L^2(0,T;H^{-s}(\Omega ))$ for $u\in \widetilde{W}_{ext}(0,T;\widetilde{H}^s(\Omega ))$.

We next show that *F* is (Fréchet) differentiable. Note that all appearing operators, up to *f*(*u*), are linear bounded operators and hence differentiable. Thus, it remains to show that *f*(*u*) is differentiable from $E_1 \rightarrow V_3$.

*Case* $2s<n$*.* By Assumption 3.4, $a\in L^{\infty }(\Omega )$ and $r>0$, we see that all conditions in Lemma 3.7 are satisfied, when $p=r+2$ and $1\le q<\infty$ is any number satisfying $r+1\le q<\infty$. Hence, the Nemytskii operator *f*(*u*) is differentiable as a map from $L^q(0,T;L^{r+2}(\Omega ))$ to $L^{\frac{q}{r+1}}(0,T;L^{\frac{r+2}{r+1}}(\Omega ))$ with differential

3.17 $$\begin{aligned} df(u)h=\partial _{\tau }f(u)h. \end{aligned}$$

Next, recall that *r* satisfies the condition (3.11) so that

$$ 1< 1+r\le 1+\frac{2s}{n-2s}=\frac{n}{n-2s}. $$

This implies

$$ 2<2+r\le 2+\frac{2s}{n-2s}=\frac{2n-2s}{n-2s}<\frac{2n}{n-2s}. $$

Thus, by the Sobolev embedding and boundedness of $\Omega$ we get

3.18 $$\begin{aligned} H^s({\mathbb {R}}^n)\hookrightarrow L^{\frac{2n}{n-2s}}(\Omega )\hookrightarrow L^{r+2}(\Omega ). \end{aligned}$$

On the other hand the conjugate exponent

$$ (r+2)'=\frac{r+2}{r+1}=1+\frac{1}{r+1} $$

fulfills

$$ 2>(r+2)'\ge 1+\frac{n-2s}{n}=\frac{2(n-s)}{n}. $$

Next, observe that

$$ \frac{2n}{n+2s}< \frac{2(n-s)}{n} \quad \Leftrightarrow \quad n^2< n^2+sn-2s^2 $$

and thus by the Sobolev embedding $L^{\frac{2n}{n+2s}}({\mathbb {R}}^n)\hookrightarrow H^{-s}(\Omega )$ we obtain

3.19 $$\begin{aligned} L^{(r+2)'}(\Omega )\hookrightarrow H^{-s}(\Omega ). \end{aligned}$$

Combining (3.18) and (3.19), we get that $u\mapsto f(u)$ is differentiable as a map from $L^q(0,T;H^s({\mathbb {R}}^n))$ to $L^{\frac{q}{r+1}}(0,T;H^{-s}(\Omega ))$. Choosing $q=2(r+1)\ge 2$ and using the embedding $\widetilde{W}(0,T;\widetilde{H}^s(U))\hookrightarrow C([0,T];\widetilde{H}^s(U))$ for any open set $U\subset {\mathbb {R}}^n$, we see that *f*(*u*) is differentiable as a map from $E_1$ to $V_3$. Next, we assert that the differential, given by (3.17), is continuous as a map from $E_1$ to $L(E_1,V_3)$. This then establishes that *f* is $C^1$ as a map from $E_1$ to $V_3$. By Lemma 3.6 with $\alpha =r$, we know that $\partial _\tau f (u)$ is continuous as a map from $L^q(0,T;L^{r+2}(\Omega ))$ to $L^{\frac{q}{r}}(0,T;L^{\frac{r+2}{r}}(\Omega ))$, when $q\ge r$. Now, let us choose *q* such that $q\ge 2\max (r,1)$ and observe that by Assumption 3.4 there holds

$$ \frac{r+2}{r}=1+\frac{2}{r}\in \left[ n/s-1,\infty \right) . $$

Therefore, we can define

3.20 $$\begin{aligned} \frac{1}{p}=\frac{n+2s}{2n}-\frac{r}{r+2}>0. \end{aligned}$$

One may observe that

$$ \frac{n+2s}{2n}-\frac{s}{n-s}\ge \frac{n-2s}{2n} $$

and hence one has

3.21 $$\begin{aligned} \frac{1}{p}\ge \frac{n+2s}{2n}-\frac{s}{n-s}\ge \frac{n-2s}{2n}. \end{aligned}$$

Now let $u_k\in E_1$, $k\in {\mathbb {N}}$, converge to some $u\in E_1$ and fix some functions $v\in E_1$, $\psi \in L^2(0,T;\widetilde{H}^s(\Omega ))$. Then by Hölders and Sobolev’s inequality we can estimate

$$ \begin{aligned}&|\langle (\partial _\tau f(u_k)-\partial _\tau f(u))v,\psi \rangle |\\&\quad \lesssim \Vert (\partial _\tau f(u_k)-\partial _\tau f(u))v\Vert _{L^2(0,T;L^{\frac{2n}{n+2s}}(\Omega ))}\Vert \psi \Vert _{L^2(0,T;L^{\frac{2n}{n-2s}}(\Omega ))}\\&\quad \overset{(3.20)}{\lesssim } \Vert \partial _\tau f(u_k)-\partial _\tau f(u)\Vert _{L^2(0,T;L^{\frac{r+2}{r}}(\Omega ))}\Vert v\Vert _{L^{\infty }(0,T;L^p(\Omega ))}\Vert \psi \Vert _{L^2(0,T;\widetilde{H}^s(\Omega ))}\\&\quad \overset{(3.21)}{\lesssim } \Vert \partial _\tau f(u_k)-\partial _\tau f(u)\Vert _{L^2(0,T;L^{\frac{r+2}{r}}(\Omega ))}\Vert v\Vert _{L^{\infty }(0,T;L^{\frac{2n}{n-2s}}(\Omega ))}\Vert \psi \Vert _{L^2(0,T;\widetilde{H}^s(\Omega ))}\\&\quad \lesssim \Vert \partial _\tau f(u_k)-\partial _\tau f(u)\Vert _{L^2(0,T;L^{\frac{r+2}{r}}(\Omega ))}\Vert v\Vert _{L^{\infty }(0,T;H^s({\mathbb {R}}^n))}\Vert \psi \Vert _{L^2(0,T;\widetilde{H}^s(\Omega ))}\\&\quad \lesssim \Vert \partial _\tau f(u_k)-\partial _\tau f(u)\Vert _{L^2(0,T;L^{\frac{r+2}{r}}(\Omega ))}\Vert v\Vert _{E_1}\Vert \psi \Vert _{L^2(0,T;\widetilde{H}^s(\Omega ))}\\&\quad \lesssim \Vert \partial _\tau f(u_k)-\partial _\tau f(u)\Vert _{L^{\frac{q}{r}}(0,T;L^{\frac{r+2}{r}}(\Omega ))}\Vert v\Vert _{E_1}\Vert \psi \Vert _{L^2(0,T;\widetilde{H}^s(\Omega ))} \end{aligned} $$

This shows that

3.22 $$\begin{aligned} \Vert \partial _\tau f(u_k)-\partial _\tau f(u)\Vert _{L(E_1,V_3)}\lesssim \Vert (\partial _\tau f(u_k)-\partial _\tau f(u))\Vert _{L^{\frac{q}{r}}(0,T;L^{\frac{r+2}{r}}(\Omega ))}. \end{aligned}$$

Now, by the continuity of $\partial _\tau f(u)$ from $L^q(0,T;L^{r+2}(\Omega ))$ to $L^{\frac{q}{r}}(0,T;L^{\frac{r+2}{r}}(\Omega ))$ and the embedding $E_1\hookrightarrow L^{\bar{p}}(0,T;H^s({\mathbb {R}}^n))$ for any $1\le \bar{p}\le \infty$, we see that (3.22) goes to zero as $k\rightarrow \infty$ and hence the differential is continuous as we wanted to show.

*Case* $2s\ge n$*.* After recalling that in this case we have $H^s({\mathbb {R}}^n)\hookrightarrow L^p(\Omega )$ for any $2\le p<\infty$ and $L^q(\Omega )\hookrightarrow H^{-s}(\Omega )$ for any $1<q\le 2$ (see [28] for supercritical Sobolev embedding), one can argue similarly as in the subcritical case $2s<n$.

Hence, *F* is a $C^1$ map. Next note that $F(0,0)=0$ . Now, the derivative of *F* at the origin in the *u*-variable is

$$ \partial _u F(0,0)v = (v(0),\partial _t v(0),v|_{(\Omega _e)_T}, (\partial _t^2+(-\Delta )^s\partial _t+(-\Delta )^s+\partial _\tau f(0))v) $$

for $v\in E_1$. This map is a linear, bounded and invertible operator from $E_1\rightarrow \prod _{j=0}^3 V_j$. To see this, consider the problem

$$\begin{aligned} {\left\{ \begin{array}{ll} ( \partial _t^2 +(-\Delta )^s\partial _t+ (-\Delta )^s + \partial _\tau f(0))v=h & \text { in }\Omega _T\\ v=\psi & \text { in } (\Omega _e)_T,\\ v=v_0,\quad \partial _tv=v_1 & \text { in }\Omega \end{array}\right. } \end{aligned}$$

for $v_0\in \widetilde{H}^s(\Omega )$, $v_1\in L^2(\Omega )$, $\psi \in W^s_{rest}((\Omega _e)_T)$ and $h\in L^2(0,T;H^{-s}( \Omega ))$. The well-posedness of this problem follows from Corollary 3.2.

Now, the implicit function theorem on Banach spaces [1, Theorem 2.3] yields that there exist neighborhoods $U_0\subset E_0$, $U_1\subset E_1$ containing the origin and a map $S\in C^1(U_0,E_1)$ such that

(i) $$F(\varphi , S(\varphi ))=0$$ for all $$\varphi \in U_0$$,
(ii) $F(\varphi ,u)=0$ for some $(\varphi ,u)\in U_0\times U_1$, then $u=S(\varphi )$.

One easily sees that $u=S(\varphi )$, for $\varphi \in U_0$, satisfies the conditions (i)–(iii). $\square$

## Inverse problem for the linear viscous wave equation

In this section we move on to the inverse problem for the viscous wave equation with linear perturbations. First in Sect.  4.1 we introduce rigorously the corresponding DN map (Definition 4.1) and then prove the Runge approximation (Proposition 4.2) in $L^2(0,T;\widetilde{H}^s(\Omega ))$. Then in Sect. 4.2 we present the proof of Theorem 1.1 after establishing a suitable integral identity in Lemma 4.3.

### DN map and Runge approximation for the linear problem

#### Definition 4.1

Let $\Omega \subset {\mathbb {R}}^n$ be a bounded Lipschitz domain, $T>0$ and $s>0$. Suppose that the (real valued) function $q\in L^1_{loc}(\Omega _T)$ satisfies the conditions in Theorem 3.1. Then we define the *Dirichlet to Neumann map* $\Lambda _q$ by

$$\begin{aligned} \langle \Lambda _q\varphi ,\psi \rangle =\int _{{\mathbb {R}}^n_T} (-\Delta )^{s/2} u (-\Delta )^{s/2} \psi \,dxdt+\int _{{\mathbb {R}}^n_T}(-\Delta )^{s/2}\partial _t u(-\Delta )^{s/2}\psi \,dxdt \end{aligned}$$

for all $\varphi ,\psi \in C_c^{\infty }((\Omega _e)_T)$. Here, $u\in \widetilde{W}_{ext}(0,T;\widetilde{H}^s(\Omega ))$ denotes the unique solution of

$$ {\left\{ \begin{array}{ll} \left( \partial _t^2 +(-\Delta )^s\partial _t +(-\Delta )^s+q\right) u=0 & \text { in }\Omega _T \\ u=\varphi & \text { in }(\Omega _e)_T, \\ u(0)=0,\quad \partial _t u(0)=0 & \text { in }\Omega . \end{array}\right. } $$

#### Proposition 4.2

(Runge approximation) Let $\Omega \subset {\mathbb {R}}^n$ be a bounded Lipschitz domain, $W\subset \Omega _e$ a given measurement set, $T>0$ and $s>0$ a non-integer. Suppose that the (real valued) function $q\in L^1_{loc}(\Omega _T)$ satisfies the conditions in Theorem 3.1. Consider the *Runge set*

$$\begin{aligned} \mathcal {R}_W:=\left\{ u_{\varphi }-\varphi \, : \, \varphi \in C^\infty _c (W_T) \right\} , \end{aligned}$$

where $u_{\varphi }\in \widetilde{W}_{ext}(0,T;\widetilde{H}^s(\Omega ))$ is the unique solution to

$$ {\left\{ \begin{array}{ll} (\partial _t^2 +(-\Delta )^s\partial _t+(-\Delta )^s + q)u = 0 & \text { in }\Omega _T \\ u=\varphi & \text { in }(\Omega _e)_T, \\ u(0)=0, \quad \partial _t u(0)=0 & \text { in }\Omega . \end{array}\right. } $$

Then $\mathcal {R}_W$ is dense in $L^2(0,T;\widetilde{H}^s(\Omega ))$.

#### Proof

Since $\mathcal {R}_W\subset L^2(0,T;\widetilde{H}^s(\Omega ))$ is a subspace it is enough by the Hahn–Banach theorem to show that if $F\in L^2(0,T;H^{-s}(\Omega ))$ vanishes on $\mathcal {R}_W$, then $F=0$. Hence, choose any $F\in L^2(0,T;H^{-s}(\Omega ))$ and assume that

$$\begin{aligned} \langle F,u_{\varphi }-\varphi \rangle =0\quad \text {for all}\quad \varphi \in C_c^{\infty }(W_T). \end{aligned}$$

Next, let $w_F\in \widetilde{W}(0,T;\widetilde{H}^s(\Omega ))$ be the unique solution to the adjoint equation

$$\begin{aligned} {\left\{ \begin{array}{ll} (\partial _t^2 -(-\Delta )^s\partial _t+(-\Delta )^s+q)w = F& \text { in }\Omega \times (0,T)\\ w=0 & \text { in }\Omega _e\times (0,T),\\ w(T)=0, \quad \partial _t w(T)=0 & \text { in }\Omega , \end{array}\right. } \end{aligned}$$

which exists by Theorem 3.1 with *q* replaced by $q^\star$ in (3.1) and a subsequent time reversal of the solution. Next, let us note that the integration by parts formula gives us

4.1 $$\begin{aligned} \begin{aligned} \int _0^T \langle \partial _t^2 v,w\rangle \,dt&=\int _0^T\langle \partial _t^2 w,v\rangle \,dt +\langle \partial _t v(T),w(T)\rangle -\langle \partial _t w(T),v(t)\rangle \\&\quad -(\langle \partial _t v(0),w(0)\rangle -\langle \partial _t w(0),v(0)\rangle ) \end{aligned} \end{aligned}$$

and

4.2 $$\begin{aligned} \begin{aligned}&\int _0^T \langle (-\Delta )^{s/2}\partial _t v,(-\Delta )^{s/2}w\rangle \,dt=-\int _0^T \langle (-\Delta )^{s/2}v,(-\Delta )^{s/2}\partial _t w\rangle \,dt\\&\quad +\langle (-\Delta )^{s/2}v(T),(-\Delta )^{s/2}w(T)\rangle -\langle (-\Delta )^{s/2}v(0),(-\Delta )^{s/2}w(0)\rangle \end{aligned} \end{aligned}$$

for all $v,w\in \widetilde{W}(0,T;\widetilde{H}^s(\Omega ))$. By a density argument, the PDEs for $u-\varphi$ and $w_F$ hold in the $L^2(0,T;H^{-s}(\Omega ))$ sense, with the help of (4.1), (4.2) and the vanishing initial and terminal conditions, respectively, we may compute

$$ \begin{aligned} 0&=\left\langle F,u_{\varphi }-\varphi \right\rangle \\&=\left\langle (\partial _t^2 -(-\Delta )^s\partial _t+(-\Delta )^s +q)w_F,u_{\varphi }-\varphi \right\rangle \\&= \left\langle (\partial _t^2 +(-\Delta )^s\partial _t+(-\Delta )^s +q)(u_{\varphi }-\varphi ),w_F\right\rangle \\&= -\left\langle (\partial _t^2 +(-\Delta )^s\partial _t+(-\Delta )^s +q)\varphi ,w_F\right\rangle \\&=-\langle (-\Delta )^s\partial _t\varphi +(-\Delta )^s\varphi ,w_F\rangle \\&=\left\langle (-\Delta )^s(w_F-\partial _t w_F),\varphi \right\rangle , \end{aligned} $$

for all $\varphi \in C_c^{\infty }(W_T)$. This implies that $\widetilde{w}_F=w_F-\partial _tw_F\in L^2(0,T;\widetilde{H}^s(\Omega ))$ satisfies

$$ (-\Delta )^s \widetilde{w}_F=\widetilde{w}_F=0\quad \text {in}\quad W_T. $$

By the unique continuation property of the fractional Laplacian [11, Theorem 1.2], this gives $\widetilde{w}_F=0$ in ${\mathbb {R}}^n_T$. By construction we have $w_F\in H^1(0,T;\widetilde{H}^s(\Omega ))$ and hence $w_F(x,\cdot )\in H^1((0,T))$ for a.e. $x\in \Omega$. Then as $\widetilde{w}_F=0$ in ${\mathbb {R}}^n_T$ we know that $w_F(x,\cdot )$ solves

$$\begin{aligned} {\left\{ \begin{array}{ll} \partial _t w_F=w_F,\\ w_F(T)=0 \end{array}\right. } \end{aligned}$$

and hence we may conclude that $w_F(x,\cdot )=0$ for a.e. $x\in \Omega$. Thus, we deduce that $w_F=0$ and therefore it follows that $F=0$ as we wanted to show. $\square$

### Unique determination of linear perturbations

In this section we give the proof of Theorem 1.1. We first deduce a suitable integral identity, which plays the role of the Alessandrini identity in the elliptic case.

As already observed, below we will make use of the following simple fact: The function $u\in \widetilde{W}_{ext}(0,T;\widetilde{H}^s(\Omega ))$ is the unique solution of

$$\begin{aligned} {\left\{ \begin{array}{ll} (\partial _t^2 +(-\Delta )^s\partial _t+(-\Delta )^s + q)u = 0 & \text { in }\Omega _T \\ u=\varphi & \text { in }(\Omega _e)_T,\\ u(0)=0, \quad \partial _t u(0)=0 & \text { in }\Omega , \end{array}\right. } \end{aligned}$$

if and only if $u^\star \in \widetilde{W}_{ext}^{\star }(0,T;\widetilde{H}^s(\Omega ))$ is the unique solution of

$$ {\left\{ \begin{array}{ll} (\partial _t^2 -(-\Delta )^s\partial _t+(-\Delta )^s + q^\star )v = 0 & \text { in }\Omega _T \\ v=\varphi ^\star & \text { in }(\Omega _e)_T, \\ v(T)=0, \quad \partial _t v(T)=0 & \text { in }\Omega . \end{array}\right. } $$

Here, $\widetilde{W}^\star (0,T;\widetilde{H}^s(\Omega ))$ denotes the space $\widetilde{W}(0,T;\widetilde{H}^s(\Omega ))+\widetilde{W}^{\star }_*(0,T;H^s({\mathbb {R}}^n))$ with

$$ \widetilde{W}^{\star }_*(0,T;H^s({\mathbb {R}}^n))=\{v\in \widetilde{W}(0,T;H^s({\mathbb {R}}^n))\,;\,v(T)\in \widetilde{H}^s(\Omega ),\,\partial _t v(T)\in L^2(\Omega ))\}. $$

#### Lemma 4.3

(Integral identity) Let $\Omega \subset {\mathbb {R}}^n$ be a bounded Lipschitz domain, $T>0$ and $s>0$. Suppose that the (real valued) function $q\in L^1_{loc}(\Omega _T)$ satisfies the conditions in Theorem 3.1. Then there holds

$$\begin{aligned} \langle (\Lambda _{q_1}-\Lambda _{q_2^{\star }})\varphi _1,\varphi _2^{\star }\rangle =\int _{\Omega _T}(q_1-q_2^{\star })(u_1-\varphi _1)(u_2-\varphi _2)^{\star }dxdt \end{aligned}$$

for all $\varphi _1,\varphi _2\in C_c^{\infty }((\Omega _e)_T)$, where $u_j\in \widetilde{W}_{ext}(0,T;\widetilde{H}^s(\Omega ))$ is the unique solution of

4.3 $$\begin{aligned} {\left\{ \begin{array}{ll} (\partial _t^2 +(-\Delta )^s\partial _t+(-\Delta )^s + q_j)u = 0 & \text { in }\Omega _T \\ u=\varphi _j & \text { in }(\Omega _e)_T,\\ u(0)=0, \quad \partial _t u(0)=0 & \text { in }\Omega \end{array}\right. } \end{aligned}$$

for $$j=1,2$$.

#### Proof

Let $Q_1,Q_2$ be two potentials satisfying the assumptions of Theorem 3.1 and denote by $U_1,U_2^{\star }$ the unique solutions of

4.4 $$\begin{aligned} {\left\{ \begin{array}{ll} (\partial _t^2 +(-\Delta )^s\partial _t+(-\Delta )^s + Q_1)u = 0 & \text { in }\Omega _T \\ u=\varphi _1 & \text { in }(\Omega _e)_T,\\ u(0)=0, \quad \partial _t u(0)=0 & \text { in }\Omega \end{array}\right. } \end{aligned}$$

and

$$\begin{aligned} {\left\{ \begin{array}{ll} (\partial _t^2 -(-\Delta )^s\partial _t+(-\Delta )^s + Q_2)v = 0& \text { in }\Omega _T \\ v=\varphi _2^{\star } & \text { in }(\Omega _e)_T,\\ v(T)=0, \quad \partial _t v(T)=0 & \text { in }\Omega , \end{array}\right. } \end{aligned}$$

respectively. Clearly, $U_2^\star$ is the time reversal of the solution $U_2$ solving

4.5 $$\begin{aligned} {\left\{ \begin{array}{ll} (\partial _t^2 +(-\Delta )^s\partial _t+(-\Delta )^s + Q_2^\star )w = 0& \text { in }\Omega _T \\ w=\varphi _2 & \text { in }(\Omega _e)_T,\\ w(0)=0, \quad \partial _t w(0)=0 & \text { in }\Omega . \end{array}\right. } \end{aligned}$$

By (4.1), we know that there holds

4.6 $$\begin{aligned} \int _0^T \langle \partial _t^2(U_1-\varphi _1),(U_2-\varphi _2)^\star \rangle \,dt=\int _0^T \langle \partial _t^2(U_2-\varphi _2)^\star ,U_1-\varphi _1\rangle \,dt. \end{aligned}$$

Therefore, we may compute

$$\begin{aligned} \begin{aligned}&\int _{\Omega _T}(Q_1-Q_2)(U_1-\varphi _1)(U_2-\varphi _2)^\star \,dxdt\\&\quad =-\int _0^T\langle (\partial _t^2+(-\Delta )^s\partial _t +(-\Delta )^s)(U_1-\varphi _1),(U_2-\varphi _2)^\star \rangle \,dt\\&\qquad +\int _0^T\langle (\partial _t^2-(-\Delta )^s\partial _t +(-\Delta )^s)(U_2-\varphi _2)^\star ,U_1-\varphi _1\rangle \,dt\\&\qquad -\int _0^T\langle (\partial _t^2+(-\Delta )^s\partial _t +(-\Delta )^s)\varphi _1,(U_2-\varphi _2)^\star \rangle \,dt\\&\qquad +\int _0^T\langle (\partial _t^2-(-\Delta )^s\partial _t +(-\Delta )^s)\varphi _2^\star ,U_1-\varphi _1\rangle \,dt\\&\quad \overset{(4.6)}{=} -\int _0^T\langle ((-\Delta )^s\partial _t +(-\Delta )^s)(U_1-\varphi _1),(U_2-\varphi _2)^\star \rangle \,dt\\&\qquad +\int _0^T\langle (-(-\Delta )^s\partial _t +(-\Delta )^s)(U_2-\varphi _2)^\star ,U_1-\varphi _1\rangle \,dt\\&\qquad -\int _0^T\langle ((-\Delta )^s\partial _t +(-\Delta )^s)\varphi _1,(U_2-\varphi _2)^\star \rangle \,dt+\int _0^T\langle (-(-\Delta )^s\partial _t +(-\Delta )^s)\varphi _2^\star ,U_1-\varphi _1\rangle \,dt. \end{aligned} \end{aligned}$$

In the second equality sign we also used the support conditions of $\varphi _j$. As the first two terms compensate each other, using integration by parts we get

$$\begin{aligned} \begin{aligned}&\int _{\Omega _T}(Q_1-Q_2)(U_1-\varphi _1)(U_2-\varphi _2)^\star \,dxdt\\&\quad = -\int _0^T\langle ((-\Delta )^s\partial _t +(-\Delta )^s)\varphi _1,(U_2-\varphi _2)^\star \rangle \,dt+\int _0^T\langle (-(-\Delta )^s\partial _t +(-\Delta )^s)\varphi _2^\star ,U_1-\varphi _1\rangle \,dt\\&\quad =-\int _0^T\langle (-(-\Delta )^s\partial _t +(-\Delta )^s)U_2^\star ,\varphi _1\rangle \,dt+\int _0^T\langle ((-\Delta )^s\partial _t +(-\Delta )^s)U_1,\varphi _2^\star \rangle \,dt\\&\quad =-\int _0^T\langle [((-\Delta )^s\partial _t +(-\Delta )^s)U_2]^\star ,\varphi _1\rangle \,dt+\int _0^T\langle ((-\Delta )^s\partial _t +(-\Delta )^s)U_1,\varphi _2^\star \rangle \,dt\\&\quad =-\int _0^T\langle ((-\Delta )^s\partial _t +(-\Delta )^s)U_2,\varphi _1^\star \rangle \,dt+\int _0^T\langle ((-\Delta )^s\partial _t +(-\Delta )^s)U_1,\varphi _2^\star \rangle \,dt. \end{aligned} \end{aligned}$$

By recalling the definition of the DN map associated with the viscous nonlocal wave equations (4.4) and (4.5), we get

4.7 $$\begin{aligned} \begin{aligned}&\int _{\Omega _T}(Q_1-Q_2)(U_1-\varphi _1)(U_2-\varphi _2)^\star \,dxdt = \langle \Lambda _{Q_1}\varphi _1,\varphi _2^{\star }\rangle -\langle \Lambda _{Q_2^{\star }}\varphi _2,\varphi _1^{\star }\rangle . \end{aligned} \end{aligned}$$

On the one hand, choosing

$$ Q_1=Q_2=q_j $$

in (4.7) ensures that

4.8 $$\begin{aligned} \langle \Lambda _{q_j}\varphi _1,\varphi _2^\star \rangle =\langle \Lambda _{q_j^*}\varphi _2,\varphi _1^\star \rangle . \end{aligned}$$

On the other hand, taking

$$ Q_1=q_1\text { and }Q_2=q_2^\star $$

in (4.7) yields

$$ \begin{aligned}&\int _{\Omega _T}(q_1-q_2^\star )(u_1-\varphi _1)(u_2-\varphi _2)^\star \,dxdt=\langle \Lambda _{q_1}\varphi _1,\varphi _2^\star \rangle -\langle \Lambda _{q_2}\varphi _2,\varphi _1^\star \rangle , \end{aligned} $$

where we used that $U_j = u_j$, with $u_j$ denoting the solution to (4.3) for $j = 1,2$. Thus, using the identity (4.8), we deduce that

$$ \begin{aligned}&\int _{\Omega _T}(q_1-q_2^\star )(u_1-\varphi _1)(u_2-\varphi _2)^\star \,dxdt =\langle (\Lambda _{q_1}-\Lambda _{q_2})\varphi _1,\varphi _2^\star \rangle . \end{aligned} $$

Hence, we can conclude the proof. $\square$

#### Proof of Theorem 1.1 for time-reversal invariant potentials

Throughout the proof we assume that $q_1, q_2$ are time-reversal invariant. Then, by Lemma 4.3 and the condition (1.10), we get

$$ \int _{\Omega _T}(q_1-q_2)(u_1-\varphi _1)(u_2-\varphi _2)^{\star }dxdt=0, $$

where $u_j$ is the unique solution of

$$\begin{aligned} {\left\{ \begin{array}{ll} (\partial _t^2 +(-\Delta )^s\partial _t+(-\Delta )^s + q_j)u = 0& \text { in }\Omega _T \\ u=\varphi _j & \text { in }(\Omega _e)_T,\\ u(0)=0, \quad \partial _t u(0)=0 & \text { in }\Omega . \end{array}\right. } \end{aligned}$$

By the Runge approximation (Proposition 4.2), for all $\Phi _1,\Phi _2\in C_c^{\infty }(\Omega _T)$ there exist sequences $v_k^{(1)}\in \mathcal {R}_{W_1}^{(1)}$ and $v_k^{(2)}\in \mathcal {R}_{W_2}^{(2)}$ such that

$$ v_k^{(1)}\rightarrow \Phi _1\text { and }v_k^{(2)}\rightarrow \Phi _2\text { in }L^2(0,T;\widetilde{H}^s(\Omega )) $$

as $k\rightarrow \infty$. Above we used the notation

$$\begin{aligned} \mathcal {R}_{W_j}^{(j)}=\{ u^{(j)}-\varphi \, : \, \varphi \in C^\infty _c ((W_j)_T) \}, \end{aligned}$$

where $u^{(j)}\in \widetilde{W}_{ext}(0,T;\widetilde{H}^s(\Omega ))$ is the unique solution to

$$ {\left\{ \begin{array}{ll} (\partial _t^2 +(-\Delta )^s\partial _t+(-\Delta )^s + q_j)u = 0 & \text { in }\Omega _T \\ u=\varphi & \text { in }(\Omega _e)_T, \\ u(0)=0, \quad \partial _t u(0)=0 & \text { in }\Omega . \end{array}\right. } $$

Hence, we have

$$ \int _{\Omega _T}(q_1-q_2)v_k^{(1)}(v_k^{(2)})^{\star }dxdt=0 $$

for all $k\in {\mathbb {N}}$. By (3.4), we get

$$ \begin{aligned}&\left| \int _{\Omega _T}(q_1-q_2)v_k^{(1)}(v_k^{(2)})^{\star }dxdt-\int _{\Omega _T}(q_1-q_2)\Phi _1\Phi _2^{\star }dxdt\right| \rightarrow 0 \end{aligned} $$

as $k\rightarrow \infty$. This shows that

$$ \int _{\Omega _T}(q_1-q_2)\Phi _1\Phi _2^{\star }dxdt=0. $$

Hence, we may conclude that there holds $q_1=q_2$ a.e. in $\Omega _T$, which completes the proof. $\square$

#### Remark 4.4

Note that if one knows a priori that the potentials $q_j$ are bounded, then a Runge approximation in $L^2(\Omega _T)$ is enough to conclude the above uniqueness proof, but for lower regular potentials one needs the Runge approximation in $L^2(0,T;\widetilde{H}^s(\Omega ))$.

#### Proof of Theorem 1.1 for generic potentials

First, let us observe that (1.10) ensures that the function

$$ w_{1,2} := \partial _t u_{1,2} + u_{1,2} \in L^2(0,T;H^s(\mathbb {R}^n)), $$

where $u_{1,2} := u_1 - u_2 \in H^1(0,T;H^s(\mathbb {R}^n))$ and $u_j$ denotes the solution to

4.9 $$\begin{aligned} {\left\{ \begin{array}{ll} (\partial _t^2 + (-\Delta )^s \partial _t + (-\Delta )^s + q_j)u = 0 &  \text {in } \Omega _T, \\ u = \varphi &  \text {in } (\Omega _e)_T, \\ u(0)=0,\quad \partial _t u(0)=0 &  \text {in } \Omega , \end{array}\right. } \end{aligned}$$

for a fixed exterior value $\varphi \in C_c^\infty ((W_1)_T)$, satisfies

$$ (-\Delta )^s w_{1,2} = w_{1,2} \quad \text {in } (W_2)_T. $$

Hence, the UCP for the fractional Laplacian [11, Theorem 1.2] implies that $w_{1,2} = 0$ in $\mathbb {R}^n_T$. By the same argument as in the proof of Proposition 4.2, we then obtain $u_{1,2} = 0$ in $\mathbb {R}^n_T$. Since $u_j$ solves (4.9) and $u_1 = u_2$ in $\mathbb {R}^n_T$, we infer that

$$ (q_1 - q_2)(u-\varphi ) = 0 \quad \text {in } \Omega _T, $$

where we set $u := u_1 = u_2$. Repeating the arguments in the proof of Theorem 1.1 for time-reversal invariant potentials, we conclude that $q_1 = q_2$ in $\Omega _T$. $\square$

## Inverse problem for the nonlinear viscous wave equation

In this section we study the inverse problem for the viscous wave equation with nonlinear perturbations. In Sect. 5.1 we first introduce rigorously the DN map and then prove a suitable integral identity (Lemma 5.2). Then finally in Sect. 5.2 we give the proof of Theorem 1.2.

### An integral identity for the nonlinear problem

#### Definition 5.1

(*The DN map*) Let $\Omega \subset {\mathbb {R}}^n$ be a bounded Lipschitz domain, $T>0$ and $s>0$ a non-integer. Suppose that we have given a nonlinearity *f* satisfying Assumption 3.4 and *f* is $r+1$ homogeneous. Let $U_0\subset \widetilde{W}^s_{rest}((\Omega _e)_T),U_1\subset \widetilde{W}_{ext}(0,T;\widetilde{H}^s(\Omega ))$ be the neighborhoods of Theorem 3.8 such that for any $\varphi \in U_0$ the problem

5.1 $$\begin{aligned} {\left\{ \begin{array}{ll} (\partial _t^2 +(-\Delta )^s\partial _t +(-\Delta )^s)u+f(u)= 0& \text { in }\Omega _T\\ u=\varphi & \text { in }(\Omega _e)_T,\\ u(0)=0,\quad \partial _t u(0)=0 & \text { in }\Omega \end{array}\right. } \end{aligned}$$

has a unique solution $u\in U_1$. Then we define the DN map $\Lambda _{f}$ related to (5.1) by

5.2 $$\begin{aligned} \begin{aligned} \left\langle \Lambda _{f}\varphi _1,\varphi _2 \right\rangle := \int _{{\mathbb {R}}^n_T}(-\Delta )^{s/2}\,u(-\Delta )^{s/2}\varphi _2 \,dxdt+\int _{{\mathbb {R}}^n_T}(-\Delta )^{s/2}\partial _t u(-\Delta )^{s/2}\varphi _2 \,dxdt, \end{aligned} \end{aligned}$$

for all $\varphi \in U_0,\psi \in C_c^{\infty }((\Omega _e)_T)$, where $u\in U_1$ is the unique solution of (5.1) with exterior condition $\varphi =\varphi _1$ (see Theorem 3.8).

#### Lemma 5.2

Let $\Omega \subset {\mathbb {R}}^n$ be a bounded Lipschitz domain, $T>0$ and $s>0$ a non-integer. Suppose that for $j=1,2$ we have given nonlinearities $f_j$ satisfying Assumption 3.4 with $r>0$ and $a\in L^{\infty }(\Omega )$ and $f_j(0)=0$. Let $U^j_0\subset \widetilde{W}^s_{rest}((\Omega _e)_T),U^j_1\subset \widetilde{W}_{ext}(0,T;\widetilde{H}^s(\Omega ))$ be the neighborhoods of Theorem 3.8 such that for any $\varphi \in U^j_0$ the problem

5.3 $$\begin{aligned} {\left\{ \begin{array}{ll} (\partial _t^2 +(-\Delta )^s\partial _t +(-\Delta )^s)u+f_j(u)= 0& \text { in }\Omega _T\\ u=\varphi & \text { in }(\Omega _e)_T,\\ u(0)=0,\quad \partial _t u(0)=0 & \text { in }\Omega \end{array}\right. } \end{aligned}$$

has a unique solution $u\in U_1^j$. Then for all exterior conditions $\varphi _1\in U_0^1\cap U_0^2\cap C_c^{\infty }((\Omega _e)_T)$ and $\varphi _2\in C_c^{\infty }((\Omega _e)_T)$ one has

5.4 $$\begin{aligned} \left\langle \left( \Lambda _{f_1} - \Lambda _{f_2}\right) \varphi _1,\varphi _2^*\right\rangle =\int _{\Omega _T} (f_1(u_1^{(1)}) - f_2(u_1^{(2)}))(u_2-\varphi _2)^\star dxdt, \end{aligned}$$

where $u_1^{(j)}$ is the unique solution of (5.3) with $\varphi =\varphi _1$ and $u_2$ is the unique solution of the linear equation

5.5 $$\begin{aligned} {\left\{ \begin{array}{ll} \left( \partial _t^2+(-\Delta )^s\partial _t+(-\Delta )^s\right) u=0, & \text {in } \Omega _T,\\ u = \varphi _2, & \text {in } (\Omega _e)_T,\\ u(0)=\partial _t u(0) = 0,& \text {in } \Omega . \end{array}\right. } \end{aligned}$$

#### Proof

First of all note that $u=u_1^{(1)}-u_1^{(2)}\in \widetilde{W}(0,T;\widetilde{H}^s(\Omega ))$ solves

5.6 $$\begin{aligned} {\left\{ \begin{array}{ll} (\partial _t^2 +(-\Delta )^s\partial _t +(-\Delta )^s)u= -(f_1(u_1^{(1)})-f_2(u_1^{(2)}))& \text { in }\Omega _T\\ u=0& \text { in }(\Omega _e)_T,\\ u(0)=0,\quad \partial _t u(0)=0 & \text { in }\Omega . \end{array}\right. } \end{aligned}$$

Then the definition of the DN map (5.2) implies

5.7 $$\begin{aligned} \begin{aligned}&\langle (\Lambda _{f_1}-\Lambda _{f_2})\varphi _1,\varphi _2^\star \rangle \\&\quad =-\int _{0}^T\langle (-\Delta )^s(u_1^{(1)}-u_1^{(2)})+(-\Delta )^s\partial _t(u_1^{(1)}-u_1^{(2)}) ,(u_2-\varphi _2)^\star \rangle \,dt\\&\quad \quad +\int _{0}^T\langle (-\Delta )^{s/2}(u_1^{(1)}-u_1^{(2)})+(-\Delta )^{s/2}\partial _t(u_1^{(1)}-u_1^{(2)}) ,(-\Delta )^{s/2}u_2^\star \rangle \,dt\\&\quad =I_1+I_2. \end{aligned} \end{aligned}$$

As $u_1^{(1)}-u_1^{(2)}$ solves (5.6), the identity (4.1) together with the fact that $u_2$ is a solution of (5.5) implies

$$\begin{aligned} \begin{aligned} I_1&=\int _0^T\langle \partial _t^2(u_1^{(1)}-u_1^{(2)})+(f_1(u_1^{(1)})-f_2(u_1^{(2)})),(u_2-\varphi _2)^\star \rangle \,dt\\&=\int _0^T(\langle \partial _t^2(u_2-\varphi _2)^\star ,(u_1^{(1)}-u_1^{(2)})\rangle +\langle (f_1(u_1^{(1)})-f_2(u_1^{(2)})),(u_2-\varphi _2)^\star \rangle )\,dt\\&=-\int _0^T\langle (-(-\Delta )^s\partial _t +(-\Delta )^s )(u_2-\varphi _2)^\star ,u_1^{(1)}-u_1^{(2)}\rangle \,dt \\&\quad -\int _0^T\langle (\partial _t^2 -(-\Delta )^s\partial _t +(-\Delta )^s)\varphi _2^\star ,u_1^{(1)}-u_1^{(2)}\rangle \,dt\\&\quad +\int _0^T \langle (f_1(u_1^{(1)})-f_2(u_1^{(2)})),(u_2-\varphi _2)^\star \rangle )\,dt \end{aligned} \end{aligned}$$

Taking into account the support condition of $\varphi _2$, we deduce that

5.8 $$\begin{aligned} \begin{aligned} I_1&=-\int _0^T\langle (-(-\Delta )^s\partial _t +(-\Delta )^s )u_2^\star ,u_1^{(1)}-u_1^{(2)}\rangle \,dt \\&\quad +\int _0^T \langle (f_1(u_1^{(1)})-f_2(u_1^{(2)})),(u_2-\varphi _2)^\star \rangle )\,dt. \end{aligned} \end{aligned}$$

On the other hand, using integration by parts, the integral $I_2$ is given by

5.9 $$\begin{aligned} \begin{aligned} I_2&=\int _{0}^T\langle (-(-\Delta )^{s/2}\partial _t+(-\Delta )^{s/2})u_2^\star ,(-\Delta )^{s/2}(u_1^{(1)}-u_1^{(2)})\rangle \,dt \end{aligned} \end{aligned}$$

Summing up (5.8) and (5.9), we deduce from (5.7) the desired identity (5.4) and can conclude the proof. $\square$

### Unique determination of nonlinear perturbations

#### Proof of Theorem 1.2

Let $\boldsymbol{\varepsilon }=(\varepsilon _0,\varepsilon _1)$ and define

$$\begin{aligned} \varphi _1^{\boldsymbol{\varepsilon }}=\varepsilon _0\psi _0+\varepsilon _1\psi _1 \end{aligned}$$

for some $\psi _j\in C_c^{\infty }((W_1)_T)$. If $|\boldsymbol{\varepsilon }|\ll 1$, then by the integral identity (5.4) with $\varphi _1=\varphi _1^{\boldsymbol{\varepsilon }}$ of Lemma 5.2 we have

5.10 $$\begin{aligned} \left\langle \left( \Lambda _{f_1} - \Lambda _{f_2}\right) \varphi ^{\boldsymbol{\varepsilon }}_1,\varphi _2^*\right\rangle =\int _{\Omega _T} (f_1(u_{\boldsymbol{\varepsilon }}^{(1)}) - f_2(u_{\boldsymbol{\varepsilon }}^{(2)}))(u_2-\varphi _2)^\star dxdt \end{aligned}$$

for $\varphi _2\in C_c^{\infty }((W_2)_T)$, where $u_{\boldsymbol{\varepsilon }}^{(j)}$ solves

$$\begin{aligned} {\left\{ \begin{array}{ll} (\partial _t^2 +(-\Delta )^s\partial _t +(-\Delta )^s)u+f_j(u)= 0& \text { in }\Omega _T\\ u=\varphi _1^{\boldsymbol{\varepsilon }}& \text { in }(\Omega _e)_T,\\ u(0)=0,\quad \partial _t u(0)=0 & \text { in }\Omega \end{array}\right. } \end{aligned}$$

and $$u_2$$ solves

$$\begin{aligned} {\left\{ \begin{array}{ll} \left( \partial _t^2+(-\Delta )^s\partial _t+(-\Delta )^s\right) u=0, & \text {in } \Omega _T,\\ u = \varphi _2, & \text {in } (\Omega _e)_T,\\ u(0)=\partial _t u(0) = 0,& \text {in } \Omega . \end{array}\right. } \end{aligned}$$

By Theorem 3.8, we know that the solution map $U_0^j\ni \varphi \mapsto u=S_j(\varphi )$ associated to

$$\begin{aligned} {\left\{ \begin{array}{ll} (\partial _t^2 +(-\Delta )^s\partial _t +(-\Delta )^s)u+f_j(u)= 0& \text { in }\Omega _T\\ u=\varphi & \text { in }(\Omega _e)_T,\\ u(0)=0,\quad \partial _t u(0)=0 & \text { in }\Omega \end{array}\right. } \end{aligned}$$

is $C^1$ as a map from $U_0^j$ to $\widetilde{W}_{ext}(0,T;\widetilde{H}^s(\Omega ))$. In particular, we see that

$$\begin{aligned} v_j=\left. \partial _{\epsilon }\right| _{\epsilon =0}S_j(\rho +\epsilon \eta )\in \widetilde{W}_{ext}(0,T;\widetilde{H}^s(\Omega )) \end{aligned}$$

exists for any $\rho \in U_0^j$ and $\eta \in C_c^{\infty }((\Omega _e)_T)$. Moreover, from the proof of Theorem 3.8 it follows that $v_j$ solves

5.11 $$\begin{aligned} {\left\{ \begin{array}{ll} (\partial _t^2 +(-\Delta )^s\partial _t +(-\Delta )^s+\partial _{\tau }f_j(S_j(\rho )))v= 0& \text { in }\Omega _T\\ v=\eta & \text { in }(\Omega _e)_T,\\ v(0)=0,\quad \partial _t v(0)=0 & \text { in }\Omega . \end{array}\right. } \end{aligned}$$

Using these observations we deduce from (5.10) that there holds

5.12 $$\begin{aligned} \begin{aligned}&\left. \partial _{\varepsilon _1}\right| _{\varepsilon _1=0}\langle (\Lambda _{f_1}-\Lambda _{f_2})\varphi _1^{\boldsymbol{\varepsilon }},\varphi _2^\star \rangle \\&\quad =\langle \partial _{\tau }f_1(u_{\varepsilon _0}^{(1)})v_{\varepsilon _0,\psi _1}^{(1)}-\partial _{\tau }f_2(u_{\varepsilon _0}^{(2)})v_{\varepsilon _0,\psi _1}^{(2)},(u_2-\varphi _2^{\star })\rangle , \end{aligned} \end{aligned}$$

where the right hand side is the duality pairing between $L^2(0,T;\widetilde{H}^s(\Omega ))$ and $L^2(0,T;H^{-s}(\Omega ))$ and we set

$$\begin{aligned} \begin{aligned} u_{\varepsilon _0}^{(j)}&=\lim _{\varepsilon _1\rightarrow 0}u_{\boldsymbol{\varepsilon }}^{(j)}\\ v_{\varepsilon _0,\psi _1}^{(j)}&=\left. \partial _{\varepsilon _1}\right| _{\varepsilon _1=0}u_{\boldsymbol{\varepsilon }}^{(j)}. \end{aligned} \end{aligned}$$

By the continuity of the solution map and (5.11), it follows that $u_{\varepsilon _0}^{(j)}$ and $v_{\varepsilon _0,\psi _1}^{(j)}$ solve

$$\begin{aligned} {\left\{ \begin{array}{ll} (\partial _t^2 +(-\Delta )^s\partial _t +(-\Delta )^s)u+f_j(u)= 0& \text { in }\Omega _T\\ u=\varepsilon _0\psi _0& \text { in }(\Omega _e)_T,\\ u(0)=0,\quad \partial _t u(0)=0 & \text { in }\Omega \end{array}\right. } \end{aligned}$$

and

$$\begin{aligned} {\left\{ \begin{array}{ll} (\partial _t^2 +(-\Delta )^s\partial _t +(-\Delta )^s+\partial _{\tau }f_j(u_{\varepsilon _0}^{(j)}))v= 0& \text { in }\Omega _T\\ v=\psi _1& \text { in }(\Omega _e)_T,\\ v(0)=0,\quad \partial _t v(0)=0 & \text { in }\Omega , \end{array}\right. } \end{aligned}$$

respectively. Next note that by the homogenity of $\partial _\tau f_j$ and arguing as above, one has

5.13 $$\begin{aligned} \varepsilon _0^{-1}u_{\varepsilon _0}^{(j)}\rightarrow v_0=\left. \partial _{\varepsilon _0}\right| _{\varepsilon _0=0}u_{\varepsilon _0}^{(j)} \end{aligned}$$

as $\varepsilon _0\rightarrow 0$ in $\widetilde{W}_{ext}(0,T;\widetilde{H}^s(\Omega ))$ and in particular in $L^q(0,T;H^s({\mathbb {R}}^n))$ for any $1\le q\le \infty$. Moreover, $v_0$ is the unique solution of

$$\begin{aligned} {\left\{ \begin{array}{ll} (\partial _t^2 +(-\Delta )^s\partial _t +(-\Delta )^s)v= 0& \text { in }\Omega _T\\ v=\psi _0& \text { in }(\Omega _e)_T,\\ v(0)=0,\quad \partial _t v(0)=0 & \text { in }\Omega . \end{array}\right. } \end{aligned}$$

Next, we show the following assertion.

#### Claim 5.3

Let $w_{\varepsilon _0,\psi _1}^{(j)}$ be defined by

$$\begin{aligned} w_{\varepsilon _0,\psi _1}^{(j)}=v_{\varepsilon _0,\psi _1}^{(j)}-\psi _1\in \widetilde{W}(0,T;\widetilde{H}^s(\Omega )). \end{aligned}$$

Then there exists $w_1\in \widetilde{W}(0,T;\widetilde{H}^s(\Omega ))$ such that

(i) $$w_{\varepsilon _0,\psi _1}^{(j)}\rightharpoonup w_1$$ in $$\widetilde{W}(0,T;\widetilde{H}^s(\Omega ))$$,
(ii) $w_{\varepsilon _0,\psi _1}^{(j)}\rightarrow w_1$ in $C([0,T];\widetilde{H}^t(\Omega ))$ for any $0\le t<s$.

Moreover, $v_1=w_1+\psi _1$ is the unique solution of

5.14 $$\begin{aligned} {\left\{ \begin{array}{ll} (\partial _t^2 +(-\Delta )^s\partial _t +(-\Delta )^s)v= 0& \text { in }\Omega _T\\ v=\psi _1& \text { in }(\Omega _e)_T,\\ v(0)=0,\quad \partial _t v(0)=0 & \text { in }\Omega . \end{array}\right. } \end{aligned}$$

#### Proof of Claim 5.3

Let us start by observing that $w_{\varepsilon _0,\psi _1}^{(j)}$ is the unique solution of

5.15 $$\begin{aligned} {\left\{ \begin{array}{ll} (\partial _t^2 +(-\Delta )^s\partial _t +(-\Delta )^s+\partial _{\tau }f_j(u_{\varepsilon _0}^{(j)}))w= -((-\Delta )^s\partial _t+(-\Delta )^s)\psi _1& \text { in }\Omega _T\\ w=0& \text { in }(\Omega _e)_T,\\ w(0)=0,\quad \partial _t w(0)=0 & \text { in }\Omega . \end{array}\right. } \end{aligned}$$

Now, we show that the function

$$ q: =\partial _{\tau }f_j(u_{\varepsilon _0}^{(j)})\in L^1_{loc}(\Omega _T) $$

satisfies the conditions (i) and (ii) of Theorem 3.1.

(i): If $2s\ge n$, then the integrability is clear as $H^s({\mathbb {R}}^n)\hookrightarrow L^p({\mathbb {R}}^n)$ for any $2\le p<\infty$. Hence, we can assume without loss of generality that $2s<n$. In this case the condition

$$ 0<r\le \frac{2s}{n-2s} $$

guarantees that

$$ \frac{n}{s}\le \frac{2n}{r(n-2s)}. $$

Therefore, we may estimate

5.16 $$\begin{aligned} \begin{aligned} \Vert \partial _\tau f_j(u_{\varepsilon _0}^{(j)})\Vert _{L^{n/s}(\Omega )}&\lesssim \Vert \partial _\tau f_j(u_{\varepsilon _0}^{(j)})\Vert _{L^{\frac{2n}{r(n-2s)}}(\Omega )}\lesssim \Vert u_{\varepsilon _0}^{(j)}\Vert ^r_{L^{\frac{2n}{n-2s}}(\Omega )}\\&\lesssim \Vert u_{\varepsilon _0}^{(j)}\Vert ^r_{H^s({\mathbb {R}}^n)}<\infty . \end{aligned} \end{aligned}$$

Here, we are using that $\partial _\tau f_j (x,\tau )$ is *r* homogeneous in the second variable, since $f_j$ is $(r+1)$ homogeneous. As $u_{\varepsilon _0}^{(j)}\in L^{\infty }(0,T;H^s({\mathbb {R}}^n))$ this shows that $\partial _\tau f_j(u_{\varepsilon _0}^{(j)})\in L^{\infty }(0,T;L^{n/s}(\Omega ))$ as we wanted to prove.

(ii): Next, note that

$$ u_{\varepsilon _0}^{(j)}=u_{\varepsilon _0}^{(j)}-\varepsilon _0\psi _0\text { in }\Omega _T. $$

By Lemma 2.9 and the Sobolev embedding, we know that

$$\begin{aligned} u_{\varepsilon _0}^{(j)}-\varepsilon _0\psi _0\in C([0,T];\widetilde{H}^s(\Omega ))\hookrightarrow C([0,T];L^{\bar{p}}(\Omega )). \end{aligned}$$

for all $\bar{p}$ satisfying

$$ {\left\{ \begin{array}{ll} 1\le \bar{p}\le \frac{2n}{n-2s},& \text { for }2s<n \\ 1\le \bar{p}<\infty ,& \text { for }2s=n \\ 1\le \bar{p}\le \infty ,& \text { for }2s>n. \end{array}\right. } $$

But then the continuity of

$$ t\mapsto \int _{\Omega }\partial _\tau f_j(u_{\varepsilon _0}^{(j)})\varphi \,dx, $$

for fixed $\varphi \in C_c^{\infty }(\Omega )$, is an immediate consequence of Hölder’s inequality, the fact that $\partial _\tau f (u)$ is continuous as a map from $L^{r+2}(\Omega )$ to $L^{\frac{r+2}{r}}(\Omega )$ and that there holds

$$ 2+r<\frac{2n}{n-2s}\text { for }2s<n. $$

This establishes the condition (ii).

Therefore, $q=\partial _{\tau }f_j(u_{\varepsilon _0}^{(j)})$ satisfies all necessary conditions in Theorem 3.1, where

5.17 $$\begin{aligned} {\left\{ \begin{array}{ll} p=n/s,& \text { for }2s<n\\ 2<p<\infty ,& \text { for }2s=n\\ 2\le p\le \infty ,& \text { for }2s>n, \end{array}\right. } \end{aligned}$$

and we can apply the energy identity of that theorem to obtain

5.18 $$\begin{aligned} \begin{aligned}&\Vert \partial _t w_{\varepsilon _0,\psi _1}^{(j)}(t) \Vert _{L^2(\Omega )}^2+ \Vert (-\Delta )^{s/2}w_{\varepsilon _0,\psi _1}^{(j)}(t)\Vert _{L^2({\mathbb {R}}^n)}^2+ 2\Vert (-\Delta )^{s/2}\partial _t w_{\varepsilon _0,\psi _1}^{(j)}\Vert _{L^2({\mathbb {R}}^n_t)}^2\\&\quad =-2\int _0^t\langle ((-\Delta )^s\partial _t+(-\Delta )^s)\psi _1(\sigma ),\partial _t w_{\varepsilon _0,\psi _1}^{(j)} (\sigma )\rangle \,d\sigma -2\langle \partial _{\tau }f_j(u_{\varepsilon _0}^{(j)}) w_{\varepsilon _0,\psi _1}^{(j)},\partial _t w_{\varepsilon _0,\psi _1}^{(j)} \rangle _{L^2(\Omega _t)}. \end{aligned} \end{aligned}$$

By (3.4), we know that there holds

$$\begin{aligned} \left| \langle \partial _{\tau }f_j(u_{\varepsilon _0}^{(j)}) u,v\rangle _{L^2(\Omega )} \right| \le C\Vert \partial _{\tau }f_j(u_{\varepsilon _0}^{(j)}(t))\Vert _{L^p(\Omega )}\Vert u\Vert _{\widetilde{H}^s(\Omega )}\Vert v\Vert _{L^2(\Omega )} \end{aligned}$$

for all $u,v\in \widetilde{H}^s(\Omega )$. Hence, the last term in the second line of (5.18) can be estimate as

5.19 $$\begin{aligned} \begin{aligned}&\left| \langle \partial _{\tau }f_j(u_{\varepsilon _0}^{(j)}) w_{\varepsilon _0,\psi _1}^{(j)},\partial _t w_{\varepsilon _0,\psi _1}^{(j)} \rangle _{L^2(\Omega _t)}\right| \\&\quad \le C\int _0^t \Vert \partial _{\tau }f_j(u_{\varepsilon _0}^{(j)}(\sigma ))\Vert _{L^p(\Omega )}(\Vert w_{\varepsilon _0,\psi _1}^{(j)}(\sigma )\Vert _{\widetilde{H}^s(\Omega )}^2+\Vert \partial _t w_{\varepsilon _0,\psi _1}^{(j)}(\sigma )\Vert _{L^2(\Omega )}^2)\,d\sigma \end{aligned} \end{aligned}$$

for some constant $C>0$ independent of *T*. On the other hand, the first term in the second line of (5.18) can be estimated as

5.20 $$\begin{aligned} \begin{aligned}&\left| \int _0^t\langle ((-\Delta )^s\partial _t+(-\Delta )^s)\psi _1(\sigma ),\partial _t w_{\varepsilon _0,\psi _1}^{(j)} (\sigma )\rangle \,d\sigma \right| \\&\quad \le C\int _0^t\Vert (-\Delta )^s\partial _t+(-\Delta )^s)\psi _1(\sigma )\Vert _{H^{-s}(\Omega )}^2+\int _0^t\Vert \partial _t w_{\varepsilon _0,\psi _1}^{(j)}(\sigma )\Vert _{\widetilde{H}^s(\Omega )}^2\,d\sigma \end{aligned} \end{aligned}$$

Note that we can absorb the last term on the left hand side of (5.18). Then combining (5.18), (5.19) and (5.20), we see that the function

$$\begin{aligned} \Phi (t)=\Vert \partial _t w_{\varepsilon _0,\psi _1}^{(j)}(t)\Vert _{L^2(\Omega )}^2+\Vert (-\Delta )^{s/2} w_{\varepsilon _0,\psi _1}^{(j)}(t)\Vert _{L^2({\mathbb {R}}^n)}+\Vert (-\Delta )^{s/2}\partial _t w_{\varepsilon _0,\psi _1}^{(j)}\Vert _{L^2({\mathbb {R}}^n_t)}^2 \end{aligned}$$

satisfies $$\Phi \in L^{\infty }((0,T))$$ and

$$\begin{aligned} \Phi (t)\le C\left( \int _0^t \Vert \partial _{\tau }f_j(u_{\varepsilon _0}^{(j)}(\sigma ))\Vert _{L^p(\Omega )}\Phi (\sigma )\,d\sigma +\int _0^t\Vert (-\Delta )^s\partial _t+(-\Delta )^s)\psi _1(\sigma )\Vert _{H^{-s}(\Omega )}^2 d\sigma \right) . \end{aligned}$$

Now, using $\partial _\tau f_j(u_{\varepsilon _0}^{(j)})\in L^{\infty }(0,T;L^{p}(\Omega ))$, we deduce from Gronwall’s inequality the estimate

5.21 $$\begin{aligned} \Phi (t)\le C\Vert (-\Delta )^s\partial _t+(-\Delta )^s)\psi _1\Vert _{L^2(0,T;H^{-s}(\Omega ))}^2e^{C\Vert \partial _\tau f_j(u_{\varepsilon _0}^{(j)})\Vert _{L^1(0,T;L^p(\Omega ))}} \end{aligned}$$

for a.e. $$0\le t\le T$$.

Next recall by (3.14) that the map

5.22 $$\begin{aligned} \partial _{\tau }f:L^q(0,T;H^s({\mathbb {R}}^n))\rightarrow L^{q/r}(0,T;L^{n/s}(\Omega )) \end{aligned}$$

is continuous for any $r\le q<\infty$. As the solution map is continuous and $f_j(0)=0$, we deduce that

$$ u_{\varepsilon _0}^{(j)}\rightarrow 0\text { in }L^{\infty }(0,T;H^s({\mathbb {R}}^n)) $$

as $\varepsilon _0\rightarrow 0$. This ensures that

5.23 $$\begin{aligned} \partial _{\tau }f_j(u_{\varepsilon _0}^{(j)})\rightarrow 0\text { in }L^{q/r}(0,T;L^{n/s}(\Omega ))\hookrightarrow L^1(0,T;L^{n/s}(\Omega )) \end{aligned}$$

as $\varepsilon _0\rightarrow 0$, for any $r\le q<\infty$. Hence, by (5.21) we achieve that

5.24 $$\begin{aligned} w_{\varepsilon _0,\psi _1}^{(j)}\text { is uniformly bounded in }\widetilde{W}(0,T;\widetilde{H}^s(\Omega )). \end{aligned}$$

Note that the uniform bound of $\partial _t^2 w_{\varepsilon _0,\psi _1}^{(j)}$ in $L^2(0,T;H^{-s}(\Omega ))$ comes from the PDE (5.15), the uniform bound of $w_{\varepsilon _0,\psi _1}$ in $H^1(0,T;\widetilde{H}^s(\Omega ))$ and the estimate (5.16) (with similar estimate in the range $2s\ge n$). By the usual embeddings we get

- $w_{\varepsilon _0,\psi _1}^{(j)}$ is uniformly bounded in $L^{\infty }(0,T;\widetilde{H}^s(\Omega ))$,
- $\partial _t w_{\varepsilon _0,\psi _1}^{(j)}$ is uniformly bounded in $C([0,T];L^2(\Omega ))$.

Next, recall that $\widetilde{H}^s(\Omega )\hookrightarrow \widetilde{H}^t(\Omega )\hookrightarrow L^2(\Omega )$ for any $0<t<s$, where the first embedding is compact. Using (a), (b) and the Aubin–Lions lemma ([34, Corollary 4]) we see that

5.25 $$\begin{aligned} w_{\varepsilon _0,\psi _1}^{(1)}\text { is relatively compact in }C([0,T];\widetilde{H}^t(\Omega )) \end{aligned}$$

for any $0<t<s$. Thus, we deduce from (5.24) and (5.25) that there exists $w_1^{(j)}\in \widetilde{W}(0,T;\widetilde{H}^s(\Omega ))$ and a subsequence of $w_{\varepsilon _0,\psi _1}^{(j)}$, $\varepsilon _0>0$, such that

(I) $$w_{\varepsilon _0,\psi _1}^{(j)}\rightharpoonup w_1^{(j)}$$ in $$\widetilde{W}(0,T;\widetilde{H}^s(\Omega ))$$ as $$\varepsilon _0\rightarrow 0$$,
(II) $w_{\varepsilon _0,\psi _1}^{(j)}\rightarrow w_1^{(j)}$ in $C([0,T];\widetilde{H}^t(\Omega ))$ for all $0<t<s$ as $\varepsilon _0\rightarrow 0$.

The convergence (I) clearly implies

5.26 $$\begin{aligned} \begin{aligned} (-\Delta )^sw_{\varepsilon _0,\psi _1}^{(j)}&\rightharpoonup (-\Delta )^s w_1^{(j)}\text { in }L^2(0,T;H^{-s}(\Omega ))\\ (-\Delta )^s\partial _t w_{\varepsilon _0,\psi _1}^{(j)}&\rightharpoonup (-\Delta )^s \partial _t w_1^{(j)}\text { in }L^2(0,T;H^{-s}(\Omega )) \end{aligned} \end{aligned}$$

as $\varepsilon _0\rightarrow 0$. On the other hand, by choosing *q* sufficiently large in (5.23) we see that

5.27 $$\begin{aligned} \begin{aligned}&\left| \int _{\Omega _T} \partial _\tau f_j(u_{\varepsilon _0}^{(j)})w_{\varepsilon _0,\psi _1}^{(j)} v\,dxdt\right| \le \int _{0}^T\Vert \partial _\tau f_j(u_{\varepsilon _0}^{(j)})\Vert _{L^{n/s}(\Omega )}\Vert v\Vert _{\widetilde{H}^s(\Omega )}\Vert w_{\varepsilon _0,\psi _1}^{(j)}\Vert _{L^2(\Omega )}\,dt\\&\quad \le \Vert w_{\varepsilon _0,\psi _1}^{(j)}\Vert _{L^{\infty }(0,T;L^2(\Omega ))}\Vert \partial _\tau f_j(u_{\varepsilon _0}^{(j)})\Vert _{L^2(0,T;L^{n/s}(\Omega ))}\Vert v\Vert _{L^2(0,T;\widetilde{H}^s(\Omega ))} \end{aligned} \end{aligned}$$

for all $v\in L^2(0,T;\widetilde{H}^s(\Omega ))$. As the first factor is uniformly bounded, we get the convergence

5.28 $$\begin{aligned} \partial _\tau f_j(u_{\varepsilon _0}^{(j)})w_{\varepsilon _0,\psi _1}^{(j)}\rightharpoonup 0\text { in }L^2(0,T;H^{-s}(\Omega )) \end{aligned}$$

as $\varepsilon _0\rightarrow 0$. Again a similar argument can be used in the cases $2s\ge n$ to obtain this convergence. Using (5.26) and (5.28), we can pass to the limit in the weak formulation of (5.15) and see that $w_1^{(j)}$ solves

5.29 $$\begin{aligned} (\partial _t^2 +(-\Delta )^s\partial _t +(-\Delta )^s)w= -((-\Delta )^s\partial _t+(-\Delta )^s)\psi _1 \end{aligned}$$

in $\Omega _T$. Additionally, from the trace theorem, we infer that

5.30 $$\begin{aligned} w_1^{(j)}(0)=\partial _t w_1^{(j)}(0)=0. \end{aligned}$$

Thus, $w_1^{(j)}$ is the unique solution of

$$ {\left\{ \begin{array}{ll} (\partial _t^2 +(-\Delta )^s\partial _t +(-\Delta )^s)w= -((-\Delta )^s\partial _t+(-\Delta )^s)\psi _1& \text { in }\Omega _T \\ w=0& \text { in }(\Omega _e)_T, \\ w(0)=0,\quad \partial _t w(0)=0 & \text { in }\Omega . \end{array}\right. } $$

and in particular is independent of *j*. As the above analysis works for any subsequence of $w_{\varepsilon _0,\psi _1}^{(j)}$, one also sees that the whole sequence needs to converge to $w_1$. Therefore, we may conclude from (5.29) and (5.30) that *v* is the unique solution of (5.14) and this finishes the proof of the Claim 5.3. $\square$

Next, let us recall that by (5.12) and (1.12), we have

$$\begin{aligned} 0=\langle \partial _{\tau }f_1(u_{\varepsilon _0}^{(1)})v_{\varepsilon _0,\psi _1}^{(1)}-\partial _{\tau }f_2(u_{\varepsilon _0}^{(2)})v_{\varepsilon _0,\psi _1}^{(2)},(u_2-\varphi _2)^{\star }\rangle . \end{aligned}$$

Multiplying this identity by $\epsilon _0^{-r}$ and using the *r* homogeneity of $\partial _\tau f_j(u)$, we get

5.31 $$\begin{aligned} 0=\langle \partial _{\tau }f_1(\varepsilon _0^{-1}u_{\varepsilon _0}^{(1)})v_{\varepsilon _0,\psi _1}^{(1)}-\partial _{\tau }f_2(\varepsilon _0^{-1}u_{\varepsilon _0}^{(2)})v_{\varepsilon _0,\psi _1}^{(2)},(u_2-\varphi _2)^{\star }\rangle . \end{aligned}$$

By (5.13) and (5.22), we know that

5.32 $$\begin{aligned} \partial _{\tau }f_j(\varepsilon _0^{-1}u_{\varepsilon _0}^{(j)})\rightarrow \partial _\tau f_j(v_0)\text { in }L^{q/r}(0,T;L^{n/s}(\Omega )) \end{aligned}$$

as $\varepsilon _0\rightarrow 0$ for any $q\ge \max (1,r)$. If we choose *q* such that $q\ge \max (1,2r)$, then the computation in (5.27) shows that

$$ \begin{aligned}&\left| \int _{\Omega _T} \partial _\tau f_j(\varepsilon _0^{-1}u_{\varepsilon _0}^{(j)})w_{\varepsilon _0,\psi _1}^{(j)} \eta \,dxdt\right| \\&\le \Vert w_{\varepsilon _0,\psi _1}^{(j)}\Vert _{L^{\infty }(0,T;L^2(\Omega ))}\Vert \partial _\tau f_j(\varepsilon _0^{-1}u_{\varepsilon _0}^{(j)})\Vert _{L^2(0,T;L^{n/s}(\Omega ))}\Vert \eta \Vert _{L^2(0,T;\widetilde{H}^s(\Omega ))}\\&\le C\Vert w_{\varepsilon _0,\psi _1}^{(j)}\Vert _{L^{\infty }(0,T;L^2(\Omega ))}\Vert \partial _\tau f_j(\varepsilon _0^{-1}u_{\varepsilon _0}^{(j)})\Vert _{L^{q/r}(0,T;L^{n/s}(\Omega ))}\Vert \eta \Vert _{L^2(0,T;\widetilde{H}^s(\Omega ))} \end{aligned} $$

for any $\eta \in L^2(0,T;\widetilde{H}^s(\Omega ))$. Using this estimate, the convergence (ii) and (5.32), we deduce that

5.33 $$\begin{aligned} \int _{\Omega _T} \partial _\tau f_j(\varepsilon _0^{-1}u_{\varepsilon _0}^{(j)})w_{\varepsilon _0,\psi _1}^{(j)} \eta \,dxdt\rightarrow \int _{\Omega _T} \partial _\tau f_j(v_0)w_1 \eta \,dxdt \end{aligned}$$

for any $\eta \in L^2(0,T;\widetilde{H}^s(\Omega ))$. In particular, (5.33) and the splitting $v_{\varepsilon _0,\psi _1}^{(j)}=w_{\varepsilon _0,\psi _1}^{(j)}+\psi _1$ allows us to pass to the limit in (5.31), which gives

5.34 $$\begin{aligned} 0=\langle (\partial _{\tau }f_1(v_0)-\partial _{\tau }f_2(v_0))v_1,(u_2-\varphi _2)^{\star }\rangle . \end{aligned}$$

Now, let $\Psi _j\in C_c^{\infty }(\Omega _T)$, $j=0,1,2$, be given functions and choose according to the Runge approximation (Proposition 4.2) the following sequences:

(A) $v^k_0-\psi _0^k\in L^2(0,T;\widetilde{H}^s(\Omega ))$, $k\in {\mathbb {N}}$, where $\psi _0^k\in C_c^{\infty }((W_1)_T)$, $v^k_0$ is the unique solution of
  $$ {\left\{ \begin{array}{ll} (\partial _t^2 +(-\Delta )^s\partial _t +(-\Delta )^s)v= 0& \text { in }\Omega _T \\ v=\psi ^k_0& \text { in }(\Omega _e)_T, \\ v(0)=0,\quad \partial _t v(0)=0 & \text { in }\Omega \end{array}\right. } $$
  and $$v^k_0-\psi _0^k\rightarrow \Psi _0$$ in $$L^2(0,T;\widetilde{H}^s(\Omega ))$$.
(B) $v^k_1-\psi _1^k\in L^2(0,T;\widetilde{H}^s(\Omega ))$, $k\in {\mathbb {N}}$, where $\psi _1^k \in C_c^{\infty }((W_1)_T)$, $v^k_1$ is the unique solution of
  $$ {\left\{ \begin{array}{ll} (\partial _t^2 +(-\Delta )^s\partial _t +(-\Delta )^s)v= 0& \text { in }\Omega _T \\ v=\psi ^k_1& \text { in }(\Omega _e)_T, \\ v(0)=0,\quad \partial _t v(0)=0 & \text { in }\Omega \end{array}\right. } $$
  and $v^k_1-\psi _1^k\rightarrow \Psi _1$ in $L^2(0,T;\widetilde{H}^s(\Omega ))$ as well as $v^k_1\rightarrow \Psi _1$ in $L^2(\Omega _T)$.
(C) $u^k_2-\varphi _2^k\in L^2(0,T;\widetilde{H}^s(\Omega ))$, $k\in {\mathbb {N}}$, where $\varphi _2^k\in C_c^{\infty }((W_2)_T)$, $u^k_2$ is the unique solution of
  $$ {\left\{ \begin{array}{ll} \left( \partial _t^2+(-\Delta )^s\partial _t+(-\Delta )^s\right) u=0, & \text {in } \Omega _T, \\ u = \varphi _2^k, & \text {in } (\Omega _e)_T, \\ u(0)=\partial _t u(0) = 0,& \text {in } \Omega \end{array}\right. } $$
  and $$u^k_2-\varphi _2^k\rightarrow \Psi _2$$ in $$L^2(0,T;\widetilde{H}^s(\Omega ))$$.

As seen in the beginning of the proof of Claim 5.3, we know that if $v\in L^{\infty }(0,T;H^s({\mathbb {R}}^n))$, then

$$ q_j=\partial _{\tau }f_j(v)\in L^{\infty }(0,T;L^p(\Omega )) $$

for some *p* satisfying the restrictions given in equation (5.17). Moreover, from (3.4) we deduce that there holds

5.35 $$\begin{aligned} \begin{aligned} \left| \langle (q_1-q_2)u,v\rangle _{L^2(\Omega _T)} \right| \le C\Vert q_1-q_2\Vert _{L^{\infty }(0,T;L^p(\Omega ))}\Vert u\Vert _{L^2(0,T;\widetilde{H}^s(\Omega ))}\Vert v\Vert _{L^2(\Omega _T)} \end{aligned} \end{aligned}$$

for all $u\in L^2(0,T;\widetilde{H}^s(\Omega ))$ and $v\in L^2(\Omega _T)$. Next, by replacing $v_1$ by $v_1^k$ and $u_2-\varphi _2$ by $u_2^k-\varphi _2^k$ in (5.34) we have

5.36 $$\begin{aligned} 0=\int _{\Omega _T} (\partial _{\tau }f_1(v_0)-\partial _{\tau }f_2(v_0))v_1^k(u^k_2-\varphi ^k_2)^{\star }\,dxdt \end{aligned}$$

for all $k\in {\mathbb {N}}$. Using (5.35), we see that in the limit $k\rightarrow \infty$ the identity (5.36) converges to

5.37 $$\begin{aligned} 0=\int _{\Omega _T} (\partial _{\tau }f_1(v_0)-\partial _{\tau }f_2(v_0))\Psi _1\Psi _2^\star \,dxdt. \end{aligned}$$

Next, we replace $v_0$ by $v_0^k$ to get

5.38 $$\begin{aligned} \begin{aligned} 0&=\int _{\Omega _T} (\partial _{\tau }f_1(v^k_0)-\partial _{\tau }f_2(v^k_0))\Psi _1\Psi _2^\star \,dxdt\\&=\int _{\Omega _T} (\partial _{\tau }f_1(v^k_0-\psi _0^k)-\partial _{\tau }f_2(v^k_0-\psi _0^k))\Psi _1\Psi _2^\star \,dxdt. \end{aligned} \end{aligned}$$

Using Lemma 3.6, we obtain that

5.39 $$\begin{aligned} \partial _\tau f_j:L^q(0,T;L^{r+2}(\Omega ))\rightarrow L^{\frac{q}{r}}(0,T;L^{\frac{r+2}{r}}(\Omega )) \end{aligned}$$

is continuous for any $r\le q<\infty$. Next, recall that we have the embedding

5.40 $$\begin{aligned} H^s({\mathbb {R}}^n)\hookrightarrow L^{r+2}(\Omega ) \end{aligned}$$

(see (3.18) for the case $2s<n$). Therefore, as by assumption we have $0<r\le 2$, we may from (5.39) and (5.40) that $\partial _\tau f_j$ is continuous as a map from $L^2(0,T;H^s({\mathbb {R}}^n))$ to $L^{\frac{2}{r}}(0,T;L^{\frac{r+2}{r}}(\Omega ))$. Hence, by Hölder’s inequality we can pass to the limit in (5.38) and get

$$ \int _{\Omega _T} (\partial _{\tau }f_1(\Psi _0)-\partial _{\tau }f_2(\Psi _0))\Psi _1\Psi _2^\star \,dxdt=0. $$

This implies that

$$ \partial _\tau f_1(x,\Psi _0(x,t))=\partial _\tau f_2(x,\Psi _0(x,t))\text { in }\Omega _T $$

for any $\Psi _0\in C_c^{\infty }(\Omega _T)$. This in turn allows us to conclude that

$$ \partial _\tau f_1(x,\rho )=\partial _\tau f_2(x,\rho )\text { for all }(x,\rho )\in \Omega \times {\mathbb {R}}. $$

Now, by the homogeneity of $f_j$ we can invoke Euler’s homogeneous function theorem to conclude that (1.13) holds and we can finish the proof of Theorem 1.2. $\square$

## Footnotes

1 For any set $A\subset {\mathbb {R}}^n$ and $T>0$, we write $A_T$ to denote the space time cylinder $A\times (0,T)$.

2 The precise definition of this space is given in Sect. 2.

3 Here, $V'$ denotes the antidual of *V*, that is the space of all antilinear continuous functionals on *V*, and the antidual of *H* is identified with *H* via Riesz’s representation theorem. Moreover, the dual of *V*, *H* are still denoted by $V^*$ and $H^*$. Recall that for any Hilbert space *X*, we have $X'=\overline{X^*}$.

4 These spaces are considered here as being complex, but later on we always assume that they consist of real valued functions.

5 Here, and throughout this work, we use the notation $\langle \cdot , \cdot \rangle$ for the natural duality pairing between the corresponding function spaces. In the present context, it denotes the pairing between $H^{-s}(\Omega )$ and $\widetilde{H}^s(\Omega )$, while later it is also used for the pairing between $L^2(0,T;H^{-s}(\Omega ))$ and $L^2(0,T;\widetilde{H}^s(\Omega ))$.
